# Are Grasses Really Useful for the Phytoremediation of Potentially Toxic Trace Elements? A Review

**DOI:** 10.3389/fpls.2021.778275

**Published:** 2021-11-24

**Authors:** Flávio Henrique Silveira Rabêlo, Jaco Vangronsveld, Alan J. M. Baker, Antony van der Ent, Luís Reynaldo Ferracciú Alleoni

**Affiliations:** ^1^Luiz de Queiroz College of Agriculture, University of São Paulo, Piracicaba, Brazil; ^2^Centre for Environmental Sciences, Hasselt University, Diepenbeek, Belgium; ^3^Department of Plant Physiology and Biophysics, Maria Curie-Skłodowska University, Lublin, Poland; ^4^Centre for Mined Land Rehabilitation, Sustainable Minerals Institute, The University of Queensland, Brisbane, QLD, Australia; ^5^School of BioSciences, The University of Melbourne, Parkville, VIC, Australia; ^6^Laboratoire Sols et Environnement, Université de Lorraine – INRAE, Nancy, France

**Keywords:** heavy metals, phytoextraction, phytofiltration, phytostabilization, Poaceae, tolerance mechanisms, toxicity, trace elements uptake

## Abstract

The pollution of soil, water, and air by potentially toxic trace elements poses risks to environmental and human health. For this reason, many chemical, physical, and biological processes of remediation have been developed to reduce the (available) trace element concentrations in the environment. Among those technologies, phytoremediation is an environmentally friendly *in situ* and cost-effective approach to remediate sites with low-to-moderate pollution with trace elements. However, not all species have the potential to be used for phytoremediation of trace element-polluted sites due to their morpho-physiological characteristics and low tolerance to toxicity induced by the trace elements. Grasses are prospective candidates due to their high biomass yields, fast growth, adaptations to infertile soils, and successive shoot regrowth after harvest. A large number of studies evaluating the processes related to the uptake, transport, accumulation, and toxicity of trace elements in grasses assessed for phytoremediation have been conducted. The aim of this review is (i) to synthesize the available information on the mechanisms involved in uptake, transport, accumulation, toxicity, and tolerance to trace elements in grasses; (ii) to identify suitable grasses for trace element phytoextraction, phytostabilization, and phytofiltration; (iii) to describe the main strategies used to improve trace element phytoremediation efficiency by grasses; and (iv) to point out the advantages, disadvantages, and perspectives for the use of grasses for phytoremediation of trace element-polluted soils.

## Introduction

The concentrations of potentially toxic trace elements in the environment have been increasing year by year due to anthropogenic activities such as mining, smelting, untreated sewage disposal, and use of pesticides and fertilizers in agriculture (Kabata-Pendias, 2011). The widespread pollution of soil, water, and air with trace elements represent one of the most severe environmental problems that can seriously affect both environmental quality and human health (Food and Agriculture Organization of the United Nations [FAO] and United Nations Environment Programme [UNEP], 2021). For this reason, many civil engineering remediation processes such as chemical extraction, ion exchange, membrane separation, and electrokinetics were developed to reduce trace element contamination in the soil (Selvi et al., 2019). Despite the achievements of these processes, they face certain disadvantages including high cost, soil disturbance, and degradation, and also potentially lead to pollution of groundwater (Selvi et al., 2019). Plant-based technologies, generally termed phytoremediation, that are considered environmentally friendly, non-invasive, energy-efficient (mainly sun-powered), and cost-effective to remediate sites with low-to-moderate concentrations of trace elements are proposed as alternative approaches (Vangronsveld et al., 2009). Nevertheless, if phytoremediation is chosen to remediate trace element pollution, the question is which kind of plant is the most suitable for this purpose? No general answer to this question can be given, as there exist different options for different cases (Tordoff et al., 2000; Vangronsveld et al., 2009).

**FIGURE 1:**
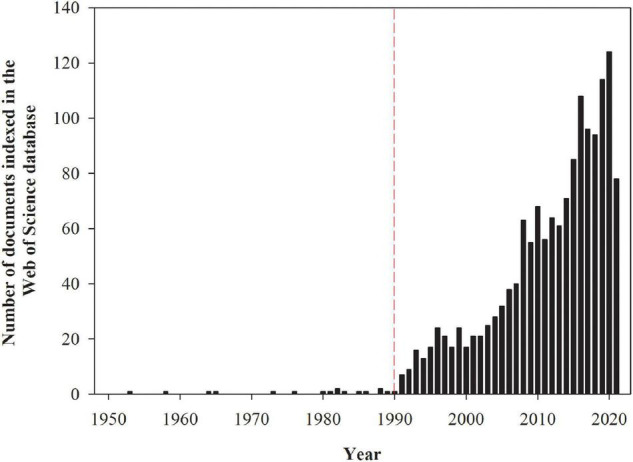
The number of documents indexed in the Web of Science database assessing the ability of grasses to survive in environments polluted by potentially toxic trace elements. The numbers presented in the figure were obtained from the search with the keywords grass and heavy metals on August 4, 2021. These numbers are subjected to changes according to keywords used for search.

**FIGURE 2:**
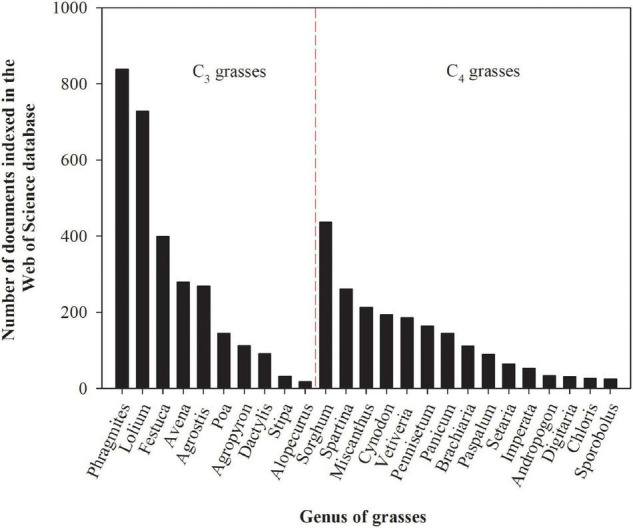
The number of documents indexed in the Web of Science database assessing the tolerance and capacity of grasses genera (C_3_ and C_4_) to remediate environments polluted by potentially toxic trace elements. The numbers presented in the figure were obtained by using the name of the genus and the keyword heavy metals as search terms on February 22, 2021. These numbers are subjected to changes according to keywords used for search.

Grasses used for biofuel or fiber production and as well as grasses normally used for animal feed (in this case the forage produced cannot be used for feedstock for animals) are potentially suitable candidates for phytostabilization, and in some cases phytoextraction, since most species have extensive root systems (which allows for the exploration of large soil volumes), high biomass yields, fast growth rates, adaptations to soil infertility, and successive shoot regrowth after harvest (Rabêlo et al., 2018a). The first studies assessing the ability of grasses to persist in environments polluted by trace elements were conducted in the early 1950s, while research in this field was boosted from the 1990s onward (Figure 1). Although most grasses have desirable characteristics for trace element phytoremediation, some genera have been more extensively studied than others (Figure 2). The greatest number of studies have been undertaken with the grasses of the genera *Phragmites*, *Lolium*, *Sorghum*, and *Festuca* are because of their large geographical distribution globally, extending from cold regions to humid wetlands in the tropics (Zeven and de Wet, 1982), and their higher tolerance to trace elements exposure. However, the potential of each grass species for trace element phytoremediation depends on several factors such as trace element bioavailability (Rabêlo et al., 2020a), the mechanisms involved in the uptake, transport, accumulation, toxicity, tolerance to each trace element (Sipos et al., 2013), and the cultivation system (Tordoff et al., 2000; Vymazal and Březinová, 2016), among other factors.

This review aims to (i) to synthesize the available information concerning the mechanisms involved in the uptake, transport, accumulation, toxicity, and tolerance to trace elements in grasses; (ii) to identify suitable grasses for phytoextraction, phytostabilization, and phytofiltration; (iii) to describe the main strategies used to improve the phytoremediation efficiency by grasses; and (iv) to point out advantages, disadvantages, and perspectives for the use of grasses for phytoremediation of polluted soils.

## Uptake, Transport, Accumulation, and Toxicity of Trace Elements in Grasses

**FIGURE 3:**
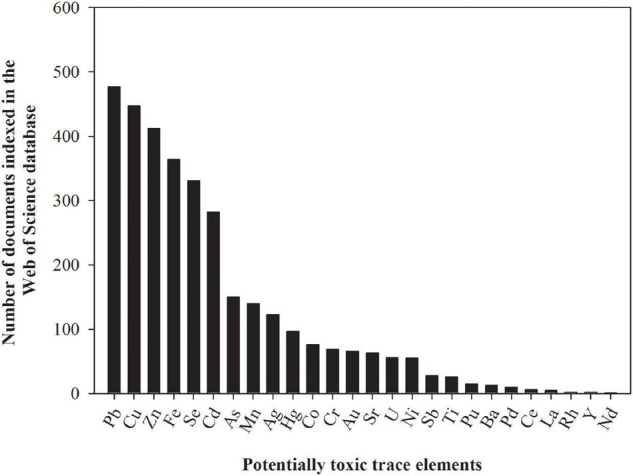
The number of documents indexed in the Web of Science database assessing the effect of potentially toxic trace elements on tolerance, survival, and/or remediation capacity of grasses. The numbers presented in the figure were obtained by using the name of the potentially toxic trace element and the keyword grass as search terms on February 23, 2021. These numbers are subjected to changes according to keywords used for search.

In the context of environmental pollution, the most important trace elements are arsenic (As), cadmium (Cd), chromium (Cr), copper (Cu), mercury (Hg), nickel (Ni), lead (Pb), and zinc (Zn) (He et al., 2015). These trace elements can be subdivided into essential (Cu, Ni, and Zn) and non-essential trace elements (As, Cd, Cr, Hg, and Pb) for plants, but both essential and non-essential trace elements can be toxic, even at low prevailing concentrations (Clemens, 2006). For this reason, many studies evaluating uptake, transport, accumulation, and toxicity of trace elements in grasses for phytoremediation applications have been conducted (Figure 3). We summarize below the main findings related to these processes for As, Cd, Cu, Hg, Ni, and Zn in grasses. Chromium and Pb are not covered in this section since both are highly insoluble (and hence unavailable for uptake) under natural conditions (Senila, 2014; Ertani et al., 2017), and most of the studies conducted with grasses used unrealistically high Cr and Pb dose rates in hydroponic solution, leading to spurious results.

### Arsenic

Non-essential elements move into and are transported through a plant along the same routes as essential elements (Clemens and Ma, 2016). Arsenic can be taken up by plants as arsenate (AsO_4_^3–^) from aerobic soil through phosphate transporters, and as arsenite (AsO_3_^3–^) from flooded soil through silicon (Si) transporters (Ma et al., 2008). Molecular studies identifying transporters involved in As uptake in monocots are scarce (except for commercial crops such as rice). To examine the contribution of cationic transporters for anions uptake, the P_1*B*_-ATPase genes family was implicated (Darabi et al., 2017). The expression of *HMA2*, *HMA5*, and *HMA7* increased in the roots of *Sorghum bicolor* in response to As exposure, suggesting that many putative pathways exist between As and Cd and P_1*B*_-ATPases to stimulate the expression or inhibition of this family. Arsenic toxicity may alter some cationic trace element transporters and induce a level of toxicity to produce synergy or may result in incompatibility between transporters when a plant is exposed to a trace element. However, it is well documented that heavy metal ATPases (HMA) transporters are involved in both Cd and Zn translocation from roots to shoots (Wong and Cobbett, 2009). After uptake, As predominantly accumulates in the plant tissues as AsO_3_^3–^, which is also the main form of As translocated from the roots to shoot (Peralta-Videa et al., 2009). The reduction of AsO_4_^3–^ to AsO_3_^3–^ is catalyzed by the enzyme arsenate reductase (AR) (EC 1.20.99.1) that is encoded by the *arsC* gene (Zhao et al., 2009). *Vetiveria zizanioides* (also known as *Chrysopogon zizanioides*) accumulated As in its shoot at a low level, which was attributed to As-induced toxicity due to the absence of both transcripts of *arsC* and AR activity (Tiwari et al., 2015). In fact, plants presenting low AR activity tend to accumulate more As in their shoots than plants with high AR activity (Dhankher et al., 2002). A rapid reduction of AsO_4_^3–^ to AsO_3_^3–^ catalyzed by AR often is followed by complexation with thiols and possibly sequestration into the root vacuoles, which results in lower mobility and translocation of As from the roots to the shoot, except in hyperaccumulators (Zhao et al., 2009). Possibly, due to these As detoxification processes, other grass species, such as *Agrostis capillaris*, *Holcus lanatus* (Dradach et al., 2020b), *Festuca rubra* (Dradach et al., 2020a), and *Pennisetum purpureum* (also known as *Cenchrus purpureus*) (Kowitwiwat and Sampanpanish, 2020), have higher As concentrations in the roots than in the shoots.

Symptoms of As-induced toxicity are closely related to changes in the integrity of plasma membranes that affect nutrient uptake and plant water status. Arsenic interacts with functional groups of enzymes, replaces phosphate groups from the ATP, and suppresses key events related to the activity of photosystem II (PSII) (for a comprehensive review see Abbas et al., 2018). An excess of As leads to the production of reactive oxygen species (ROS) in the cell which can disturb the redox balance and cause oxidative stress. The generation of ROS in plants exposed to As should be linked to the reduction of AsO_4_^3–^ to AsO_3_^3–^ (Abbas et al., 2018). The exposure to high As concentrations (10, 50, 100, and 200 μmol L^–1^) induced oxidative stress in both the roots and shoots of *V. zizanioides* after 7 and 14 days of exposure (Singh et al., 2017). Nevertheless, the low or absent AR activity in *V. zizanioides* (Tiwari et al., 2015) suggests that ROS generation in grasses can also occur from other As-induced biochemical changes. Arsenic negatively affects seed germination. Diminished seed germination in *F. rubra* exposed to As concentrations higher than 6 mg L^–1^ was reported (Vázquez de Aldana et al., 2014). Other studies describing symptoms of As-induced toxicity in grasses are presented in Table 1.

**TABLE 1:** Symptoms of toxicity induced by As, Cd, Cu, Ni, or Zn in grasses assessed for phytoremediation.

Trace elements	Grass species	Growth medium	Exposure dose	Exposure time	Changes induced by toxicity	References
As	*Brachiaria decumbens*	Soil	0, 25, 50, 200, and 800 mg kg^–1^ Na_2_HAsO_4_.7H_2_O	55 days	Decline on root and shoot biomass yield	Araújo et al., 2011
	*Pennisetum clandestinum*	Nutrient solution	0, 10, 50, 100, and 250 μmol L^–1^ Na_2_HAsO_4_	5 days	Decreased root elongation and P uptake	Panuccio et al., 2012
	*Spartina alterniflora*	Hydroponics	0.2, 0.8, and 2.0 mg L^–1^ AsO_2_^–^, AsO_4_^3–^, methyl arsonic acid or dimethyl arsinic acid	30 days	Decline on root and shoot biomass yield	Carbonell-Barrachina et al., 1998
	*Spartina patens*	Hydroponics	0.2, 0.8, and 2.0 mg L^–1^ AsO_2_^–^, AsO_4_^3–^, methyl arsonic acid or dimethyl arsinic acid	30 days	Decline on root and shoot biomass yield	Carbonell-Barrachina et al., 1998
	*Vetiveria zizanioides*	Nutrient solution	25, 50, 100, 200, 400, 800 μmol L^–1^ NaAsO_2_ or As_2_O_5_	28 days	Lipid peroxidation in roots and shoot, decline on root and shoot biomass yield, epidermal cells of root out of shape, protoplasts shrunk to a varied extent in the epidermal cell of roots	Yu et al., 2020
Cd	*Brachiaria decumbens*	Soil	0.63 and 3.6 mg kg^–1^ CdCl_2_	64 days	Disorder on nutrients uptake and N metabolism, irregular pericycle cells, oxidative stress in leaf blades, and increase in the size and number of starch grains	Rabêlo et al., 2020b
	*Lolium perenne*	Nutrient solution	1000 μmol L^–1^ CdCl_2_	3, 6, and 9 days	Reduced number of tillers and axillary buds, and decreased cytokinin biosynthesis gene expression	Niu et al., 2021
	*Panicum maximum*	Soil	0.63 and 3.6 mg kg^–1^ CdCl_2_	64 days	Decreased nutrient use efficiency, irregular pericycle cells, oxidative stress in leaf blades, and increase in the size of starch grains	Rabêlo et al., 2020b
	*Pennisetum purpureum*	Nutrient solution	20, 40, 60, 80, and 100 mg L^–1^	1, 15, 30, and 45 days	Decreased growth rate	Tananonchai and Sampanpanish, 2020
	*Pennisetum purpureum* × *Pennisetum glaucum*	Soil	0, 0.25, 0.5, 1, 2, 4, 8, and 16 mg kg^–1^ Cd	120 days	Decline on root biomass yield during elongation stage	Zhou et al., 2020
Cu	*Arundo donax*	Nutrient solution	1, 2, 3, 5, 10, and 26.8 mg L^–1^ CuSO_4_.5H_2_O	42 days	Decreased root and shoot length	Elhawat et al., 2015
	*Panicum maximum*	Nutrient solution	0.3, 100, 500, and 1000 μmol L^–1^	23 days	Decline on shoot biomass yield during growth and regrowth, decreased root growth, reduced number of leaves and tillers, lipid peroxidation in diagnostic leaves	Gilabel et al., 2014
	*Pennisetum purpureum*	Soil	50, 100, 500, 1500, 3000 mg kg^–1^ CuSO_4_.5H_2_O	60 days	Decline on biomass yield, lower chlorophyll concentration, and decreased net photosynthetic rate	Liu et al., 2009
	*Phragmites australis*	Soil	50, 100, 500, 1500, 3000 mg kg^–1^ CuSO_4_.5H_2_O	60 days	Decline on biomass yield, lower chlorophyll concentration, and decreased net photosynthetic rate	Liu et al., 2009
	*Vetiveria zizanioides*	Soil	50, 100, 500, 1500, 3000 mg kg^–1^ CuSO_4_.5H_2_O	60 days	Decline on biomass yield, lower chlorophyll concentration, and decreased net photosynthetic rate	Liu et al., 2009
Hg	*Avena sativa Chloris barbata*	Soil soil	0, 2.5, 5, 10, 20, 40, and 80 mg kg^–1^ HgCl_2_ 0.001, 0.01, 0.05, 0.1, and 0.5 mg L^–1^ HgCl_2_	5 days	Reduced stomatal conductance and net photosynthetic rate, and lipid peroxidation and lowered synthesis of proline in the leaves Decreased root elongation	Lima et al., 2021 Patra et al., 1994
	*Paspalum distichum*	Soil	0.11 and 223 mg kg^–1^ Hg	60 days	Decreased root length and root biomass, and oxidative stress in the roots	Ding et al., 2019
Ni	*Agrostis gigantea*	Nutrient solution	0 and 150 μmol L^–1^	4 days	Impaired growth root	Rauser and Winterhalder, 1985
	*Deschampsia cespitosa*	Nutrient solution	0, 50, and 1600 μmol L^–1^	4 days	Impaired growth root	Rauser and Winterhalder, 1985
	*Phalaris arundinacea*	Soil	0, 40, 80, and 160 mg kg^–1^ Ni	2 years	Decreased root and shoot biomass yield, and lower net photosynthetic rate	Korzeniowska and Stanislawska-Glubiak, 2019
	*Phalaris arundinacea*	Soil	0, 40, 80, and 160 mg kg^–1^ Ni	1 year	Decreased biomass yield	Korzeniowska et al., 2011
	*Poa compressa*	Nutrient solution	0, 50, and 150 μmol L^–1^	4 days	Impaired growth root	Rauser and Winterhalder, 1985
Zn	*Agrostis gigantea*	Nutrient solution	0 and 600 μmol L^–1^	4 days	Impaired growth root	Rauser and Winterhalder, 1985
	*Chloris barbata*	Soil	1, 5, 10, 20, and 40 mg L^–1^ ZnSO_4_.7H_2_O	5 days	Decreased root elongation	Patra et al., 1994
	*Deschampsia cespitosa*	Nutrient solution	0, 1.2, and 4 mmol L^–1^	4 days	Impaired growth root	Rauser and Winterhalder, 1985
	*Phalaris arundinacea*	Soil	0, 200, 400 and 800 mg kg^–1^ Zn	1 year	Decreased biomass yield	Korzeniowska et al., 2011
	*Poa compressa*	Nutrient solution	0, 300, 600, and 1200 μmol L^–1^	4 days	Impaired growth root	Rauser and Winterhalder, 1985

### Cadmium

Unlike the anions AsO_4_^3–^ and AsO_3_^3–^, cations such as Cd^2+^ can reach the vascular system of plants through the apoplastic and symplastic pathways (Lux et al., 2011). In an attempt to evaluate the contribution of apoplastic and symplastic pathways for Cd uptake in *Panicum maximum* (synonym *Megathyrsus maximus*), Rabêlo et al. (2017c) reported that Cd was taken up through both pathways, but that the symplastic contribution was affected by sulfur (S) supply, probably due to S-induced changes in the synthesis of phytochelatins (PCs) [(γ-Glu-Cys)_*n*_-Gly, with *n* = 2–11]. These S-rich compounds are involved in Cd^2+^ sequestration from the cytosol to the vacuole (Rabêlo et al., 2018b). From the symplastic pathway, Cd can enter root cells as Cd^2+^ through Zinc-regulated transporter/Iron-regulated transporter-like Protein (ZIP) transporters and Cd-chelates through Yellow-Stripe 1-Like (YSL) proteins, whereas transporters of the P_1*B*_-ATPases family are involved in Cd loading into the xylem (Lux et al., 2011). The genes *HMA5* and *YSL5* were upregulated in response to Cd, and both contributed to Cd transport from the symplast into the xylem in *P. americanum* × *purpureum* plants (Zhao J. et al., 2019). In *F. arundinacea* (also known as *Schedonorus arundinaceus*), the *HMA3* gene was found to play a key role in the Cd translocation from the roots to the shoots (Zhu et al., 2020). On the other hand, several transporters of the P_1*B*_-ATPases family were downregulated in the roots and shoot of *S. bicolor* exposed to 50 μmol Cd L^–1^, but *HMA8* was up-regulated at a Cd concentration of 500 μmol Cd L^–1^ in solution, leading to the conclusion that this gene has a role in the adaptation of sorghum to Cd (Zhiguo et al., 2018). These results indicate that the expression of genes related to Cd transport is tissue- and species-specific.

Cadmium concentrations in the roots and shoots are mainly determined by the Cd entry into the roots, sequestration within root vacuoles, translocation in both xylem and phloem, and dilution within the shoot due to growth (Lux et al., 2011; Clemens and Ma, 2016). Among the metabolites involved in Cd sequestration into the vacuoles, the most important are PCs that exert a great influence on Cd accumulation, mainly in the roots (Clemens, 2006). Cadmium accumulation in the roots of *P. maximum* was closely related to PCs synthesis in plants exposed to 100 or 500 μmol Cd L^–1^ in solution for 9 days (Rabêlo et al., 2018b). However, in plants that are longer exposed to Cd, binding by PCs in the roots is decreasing. In the roots of *Brachiaria decumbens* (also known as *Urochloa decumbens*) and *P. maximum* grown for 64 days in an Oxisol with 3.6 mg Cd kg^–1^ (Rabêlo et al., 2021), the PC concentrations were only slightly higher than in the unpolluted control soil. Cadmium binding by cell walls (apoplast) was probably the process that most contributed to Cd accumulation in the roots of grasses under prolonged Cd exposure (Rabêlo et al., 2021). As speculated by Clemens (2006), Cd exposure time influences the binding partners for Cd accumulation. Even facing efficient mechanisms to avoid translocation from the roots to the shoot, Cd tends to accumulate more in the shoots compared with other trace elements (Clemens and Ma, 2016), in a process that is also influenced by transpiration (Lux et al., 2011). Nevertheless, comparing a potential Cd-accumulator (*P. americanum* × *purpureum*) and a Cd-excluder (*V. zizanioides*), the transpiration was correlated to the shoot Cd concentration only in the accumulator species (Zhang et al., 2014). The positive correlation between transpiration and shoot Cd accumulation is expected to exist only in tolerant grasses since Cd accumulation often decreases the leaf area of Poaceae plants, which is lowering the transpiratory flux (Kaznina and Titov, 2014).

Cadmium-induced toxicity symptoms in grasses may result from a wide range of interactions at the cellular level (Gallego et al., 2012). Cadmium exposure (15, 30, 60, and 100 mg Cd kg^–1^ soil) led to nutritional imbalance and reduced the net photosynthetic rate in *P. americanum* × *purpureum* and *V. zizanioides* (Zhang et al., 2014). Nutritional imbalance has been identified out as one of the main causes for growth inhibition and low biomass yield in two genotypes of *P. maximum* (Rabêlo et al., 2020b). Cadmium exposure (500–2,000 μmol L^–1^ in solution) also reduced the tillering in *P. maximum*, compromising the plant capacity to produce leaves and perform photosynthesis (Rabêlo et al., 2017a). Cadmium-induced changes of the photosynthetic apparatus have been suggested as the main cause for the low biomass yield in Cd-exposed Poaceae (Kaznina and Titov, 2014). Ultrastructural changes of the chloroplast and 60% lower ribulose 1,5-bisphosphate carboxylase/oxygenase (Rubisco, EC 4.1.1.39) activity in *P. australis* exposed to 100 μmol Cd L^–1^ in a solution were reported in comparison with non-exposed controls (Pietrini et al., 2003). Most studies to date involved acute Cd exposure (i.e., high exposure dose in a short time), however, from the perspective of phytoremediation, it is necessary to better understand the consequences of chronic exposure (i.e., low exposure dose for a long time).

### Copper

Copper is taken up by transporters of the Copper Transporter (COPT) and ZIP families from the symplastic pathway, while transporters of the YSL and P_1*B*_-ATPases families are involved in Cu loading into the xylem (Andresen et al., 2018; Kumar et al., 2021). In *Brachypodium distachyon*, *COPT3* and *COPT4* are localized in the plasma membrane and are transcriptionally upregulated in both the roots and leaves by Cu deficiency, while *COPT3*, *COPT4*, and *COPT5* confer low affinity to Cu transport (Jung et al., 2014). These results differ from those reported for other plants, in which *COPT1*, *COPT2*, and *COPT6* were reported to have higher expression in the leaves, *COPT3* and *COPT5* are mainly expressed in the stems, and *COPT4* in roots (Andresen et al., 2018). It is very probable, as also mentioned for Cd, that the expression of genes related to Cu transport is specific to tissues and grass species. Due to the strong binding of Cu^2+^ to the cell walls (Kopittke et al., 2011), the contribution of the apoplastic pathway to Cu uptake is believed to be low. Probably because of the action of the negative charges of the cell wall on Cu^2+^ binding, Cu is long-distance transported as Cu^+^ bound to metallothioneins (MTs) and/or complexed with organic ligands such as histidine, nicotianamine (NA), and 2’-deoxymugineic acid (Andresen et al., 2018). However, to the best of our knowledge, there exist no studies addressing the role of organic ligands in the long-distance transport of Cu in grasses used for Cu phytoremediation.

The limited number of Cu hyperaccumulator plants suggests that most plants are highly effective at excluding Cu from uptake and that this element is preferentially accumulated in the roots (Kumar et al., 2021). *Panicum maximum* exposed to Cu concentrations ranging from 0.3 to 500 μmol L^–1^ accumulated more Cu in the roots than the shoots, but the opposite was observed when this plant was exposed to 1,000 μmol L^–1^ Cu (Gilabel et al., 2014). Similarly, the Cu concentration in *F. rubra* exposed to 5 mg Cu L^–1^ was higher in the roots than in the shoots, but the opposite was found when this plant was exposed to 10 mg Cu L^–1^ (Malagoli et al., 2014). The Cu concentration in *F. rubra* and external Cu were dose-dependent (Malagoli et al., 2014), but most likely, Cu immobilization in the roots by cell wall binding, Cu chelation by PCs, MTs, and organic acids, or Cu compartmentation in the vacuoles are not sufficient in situations of extremely high Cu concentrations in the roots, and in this case, the plant translocates a great part of the Cu that is taken up to the shoot, which can lead to plant death. Copper distribution into the different aerial tissues in grasses is controlled by the nodes through a phloem-kickback mode (for a comprehensive review see Yamaji and Ma, 2014). In this sense, high Cu concentrations in the nodes could cause Cu toxicity and alter Cu distribution between the roots and the shoots. However, this assumption needs to be carefully investigated, because plants possess some mechanisms to cope with the excess of Cu, such as (i) immobilization of Cu in the roots (e.g., binding to cell walls) avoiding Cu translocation to shoot, (ii) stimulation of the efflux pumping trace element at the plasma membranes, (iii) chelation of Cu by PCs, MTs, organic acids, or heat shock proteins, and (iv) compartmentation of Cu in the vacuoles, which also affects the capacity of Cu accumulation (Kumar et al., 2021). For instance, a genotype of *F. rubra* containing higher concentrations of malic acid also contained higher Cu concentrations in its shoots Cu (Harrington et al., 1996).

At the cellular level, excess Cu can cause toxicity due to (i) binding to sulfhydryl groups in proteins, thereby inhibiting enzyme activity or protein function, (ii) induction of the deficiency of other essential ions, (iii) impaired cell transport processes, and (iv) oxidative damages (Andresen et al., 2018; Kumar et al., 2021). Souza Junior et al. (2019) evaluated the effects of 0.3, 250, 500, and 1,000 μmol Cu L^–1^ in the leaves and other shoot tissues of *P. maximum* and observed that the highest Cu concentrations induced oxidative stress in all tissues, but Cu exposure did not lead to increased hydrogen peroxide (H_2_O_2_) concentrations. This can be explained by the fact that transition trace elements like Cu catalyze the formation of hydroxyl radicals (^⋅^OH) from the non-enzymatic chemical reaction between superoxide (O_2_^⋅–^) and H_2_O_2_ through Fenton and Haber-Weiss reactions (Kumar et al., 2021). The Cu-induced generation of ^⋅^OH can strongly affect photosynthesis by decreasing the chlorophyll concentrations and affecting the PSII reaction centers (Richards et al., 2015; Kumar et al., 2021). Indeed, lower chlorophyll concentrations after Cu-exposure were reported for *F. rubra* (Malagoli et al., 2014), while the lower electron transport rate (ETR) and quantum yield of PSII (ϕPSII) were described in *P. maximum* (Souza Junior et al., 2019). These results indicate that the Cu-induced toxicity effects on photosynthesis are similar for C_3_ and C_4_ grasses. As a result of Cu-induced physiological changes, a decline of biomass production, reduction of root growth, chlorosis, bronzing, and necrosis are the most common symptoms associated with exposure to excess Cu (Kumar et al., 2021). Some of these Cu-toxicity symptoms are described in Table 1.

### Mercury

Compared with other trace elements such as As and Cd, information about Hg uptake by plants is scarce (Clemens and Ma, 2016). It is believed that Hg is readily taken up by plant roots through a low-affinity root transporter and predominantly accumulated in the roots (Esteban et al., 2008; Chen and Yang, 2012). Mercury (Hg^2+^) import into root cells occurs possibly through Fe, Cu, or Zn transporters/channels (Chen and Yang, 2012). A kinetical study pointed out that Hg accumulation in the roots is dose-time-dependent and shows saturable behavior for the lower Hg concentrations and linear behavior for the highest ones (Esteban et al., 2008). The Hg^2+^-ion easily interacts with anionic compounds (e.g., sulfate and phosphate) forming insoluble precipitates, which limit its symplastic mobility (Chen and Yang, 2012). In *V. zizanioides* exposed to 0.8 mg L^–1^ HgCl_2_, Hg was mainly found in the root epidermis and exodermis (tissues containing high lignin), whereas trace levels of Hg were localized in the vascular bundles (Lomonte et al., 2014). Approximately 80% of the Hg trapped in the roots is bound to the cell walls (Wang and Greger, 2004). Therefore, Hg translocation from the roots to the shoots is believed to be very low (Chen and Yang, 2012; Lomonte et al., 2014). The shoot, particularly the leaf, is another important route for Hg^0^ uptake due to the industrial emissions of Hg to the air and microbial-mediated Hg emissions from soils (Chen and Yang, 2012). Indeed, the Hg concentration in the leaves of *Lolium perenne* increased linearly with the Hg concentrations in the air (De Temmerman et al., 2007). The mechanisms of how gaseous Hg enters leaves remain elusive, but the stomata may be responsible for the uptake of Hg by the leaves through gas exchange (Chen and Yang, 2012; Shahid et al., 2017). In plants exposed to Hg^0^ in the atmosphere, the Hg accumulated in the shoot does not move to the roots (Suszcynsky and Shann, 1995). This statement apparently is also applicable to grasses. Niu et al. (2013) measured the Hg concentrations in the roots and leaves of *L. perenne* exposed to gaseous Hg and/or Hg-containing soil and found that Hg originating from the soil had a significant influence on the Hg accumulation in the roots, but had no influence on the Hg accumulation in the leaves that were accumulated from the atmosphere. Niu et al. (2013) concluded that Hg presence in the leaves of grasses is mainly of atmospheric origin. In summary, Hg accumulation depends on if the plant is exposed to elemental (vapor) Hg^0^ through the shoot or Hg^2+^ ions through roots (Suszcynsky and Shann, 1995).

The toxicity due to Hg exposure is often associated with ROS generation, disturbance of almost any function in which critical or non-protected proteins are involved due to the high affinity of Hg for sulphydryl (–SH), negative effects on both light and dark reactions of photosynthesis, and inhibition of the water channels in the membrane of higher plant cells (Patra et al., 2004; Chen and Yang, 2012). The photosynthetic rate, stomatal conductance, and transpiration decreased in *Avena sativa* grown in an Oxisol (Typic Hapludox) polluted with 2.5–80 mg Hg kg^–1^ soil, but no oxidative stress in the leaves was observed (Lima et al., 2021). Possibly, the Hg concentrations in the leaves of *A. sativa* were not high enough to induce oxidative stress, or the Hg^2+^ was reduced inside the plant into Hg^0^ to avoid the binding of Hg^2+^ to –SH groups of antioxidative compounds. Both *Phragmites australis* and *Spartina alterniflora* can release Hg^0^ from their leaves and thus lower Hg toxicity, in a process dependent on Hg^2+^ reduction into Hg^0^ (Windham et al., 2001). As roots are the main site of Hg^2+^ accumulation, inhibitory effects on root growth are expected. The root elongation of *Iseilema membranaceum*, *Dichanthium sericeum*, and *Sporobolus africanus* decreased after 28 days of exposure to 5,000 mg Hg L^–1^ (Mahbub et al., 2017). More studies describing the symptoms of Hg-induced toxicity are presented in Table 1.

### Nickel

Nickel is taken up by plant roots *via* passive diffusion and active transport, but the ratio of uptake between active and passive transport varies with the species, the chemical form of Ni, and its concentration in the growth medium (Yusuf et al., 2011). Chintani et al. (2021) studied the Ni uptake by *V. zizanioides* and found that the uptake rate and efflux rate of Ni were dose-time-dependent, but no mechanisms related to Ni uptake were elucidated. To date, there is no evidence for the existence of Ni-specific transporters in plants. Nickel uptake by roots appears to occur through poorly selective cation transporters, notably members of the ZIP family that are responsible for Fe and Zn uptake (van der Pas and Ingle, 2019). In line with this hypothesis, the inhibition of Ni accumulation by Zn and Fe has been reported in Ni hyperaccumulator plants (Taylor and Macnair, 2006). After being taken up, the chelation of Ni in the cytosol by ligands is thought to be an important process in the root-to-shoot Ni transport (Yusuf et al., 2011). The preferred, but not exclusive, ligand of Ni is histidine (Andresen et al., 2018). Nicotianamine also appears to be involved as a ligand in the long-distance transport of Ni (Andresen et al., 2018). Nickel accumulation in the shoot of *L. perenne* was closely related to high xylem transport rates and citric acid concentration (Yang et al., 1997), which suggests that this organic acid is also involved in Ni translocation in grasses. Although Ni can also enter plants *via* leaves (Yusuf et al., 2011; Shahid et al., 2017), the main routes of Ni uptake and transport in plants are from the roots to the shoots through the transpiration stream *via* the xylem (Yusuf et al., 2011). A positive correlation was observed between Ni accumulation in the shoot of *Agropyron elongatum* and transpiration (Sipos et al., 2013), which suggests that Ni translocation in this species is driven mainly by transpiration. However, other processes also influence Ni translocation from the roots to the shoot in plants, such as Ni chelation. In *A. elongatum*, the Ni translocation factor (TF) remained below 0.38, regardless of the Ni concentration in the soil (Chen and Wong, 2006). A low Ni accumulation in the shoot has been associated with the absence of Ni ligands since without being chelated by ligands, Ni translocation from the roots to the shoot is expected to be retarded as xylem cell walls have a high cation exchange capability (CEC) (Yusuf et al., 2011). On the other hand, histidine could promote the root-to-shoot translocation of Ni by reducing the vacuolar sequestration of Ni in the root tissues of hyperaccumulators (van der Pas and Ingle, 2019). The same probably occurs when citric acid is functioning as a Ni ligand (Yang et al., 1997). Thus, the higher Ni accumulation in the shoots than the roots in *Cynodon dactylon* and *F. arundinacea* (Soleimani et al., 2009) could be related to a higher synthesis of histidine and/or citric acid.

The toxicity induced by Ni is mainly related to (i) the displacement of essential constituents in biomolecules, (ii) binding to essential biological functional groups of the molecules, and (iii) modification of enzyme/proteins, plasma membrane, and/or membrane transporters structure/function, which can lead to nutritional imbalance (Yusuf et al., 2011). Nickel exposure decreased both manganese (Mn) and Zn concentrations in the shoots of *L. perenne* (Khalid and Tinsley, 1980). Also, a lowered Mn concentration was reported in the shoots of Ni-exposed *Urochloa mosambicensis*, *B. decumbens*, *Chloris ventricose*, *Astrebla lappacea*, and *C. ciliaris*, as well as lower biomass yield due to Ni toxicity (Kopittke et al., 2010). Nickel accumulation limited the biomass yield of *P. maximum*, *B. brizantha*, and *B. decumbens* (Silva et al., 2017). Furthermore, Ni can disturb the balance between the formation and scavenging of ROS in plant cells (van der Pas and Ingle, 2019), resulting in oxidative stress. Nickel also damages the photosynthetic apparatus by impairing mesophyll cells and epidermal tissues, reducing chlorophyll content, and increasing the number of non-appressed lamellae (Yusuf et al., 2011). Studies describing symptoms of Ni toxicity at the molecular level in grasses are very scarce. Most studies focus on the general symptoms of Ni-toxicity, such as root growth inhibition and decreased biomass yield (Table 1).

### Zinc

Zinc is taken up from the soil solution as Zn^2+^ or complexed with organic ligands. The roots feed the shoot *via* the xylem (Broadley et al., 2007). Grasses acquire (extra?) Zn by releasing mugineic acid for ferric ion (Fe^3+^) uptake [Strategy II for iron (Fe) uptake]. This phytosiderophore chelates in addition to Fe^3+^ also Zn^2+^ (Andresen et al., 2018). Also, derivatives of mugineic acid such as 3-epi-hydroxymugineic acid are involved in Zn uptake by grasses grown in calcareous soils, as described by Cakmak et al. (1996) for *A. orientale*. The Fe^3+^-phytosiderophore transporter *YS1* also transports mugineic acid complexed with Zn^2+^ (Schaaf et al., 2004). Zinc loading into the xylem is accomplished by active Zn export out of the symplast, mediated by members of the HMA of the P_1*B*_-ATPases family (Andresen et al., 2018). Besides the symplastic pathway, Zn can be delivered extracellularly to the stelar apoplast in zones where the Casparian bands are not fully formed (Broadley et al., 2007). In the xylem, Zn can be transported in its free form or bound to organic acids. How Zn is exported from the xylem into aerial tissues is not yet completely understood, but Zn translocation from the roots to shoots involves transporters of the ZIP family (Andresen et al., 2018). In *Setaria italica* and *P. sumatrense*, seven ZIP genes (*ZIP1-ZIP7*) are involved in Zn uptake, translocation, and storage (Alagarasan et al., 2017). This result is quite interesting if we consider that most of the Zn taken up by roots of grasses are preferentially distributed to the nodes and then delivered to developing tissues exclusively through the phloem, where the Zn loading into phloem is mediated by *HMA2* transporters (Yamaji and Ma, 2014). In this sense, there is an important contribution not only of xylem but also phloem for Zn distribution in the shoots of grasses.

After being taken up, Zn can be accumulated in the roots in processes that depend on the synthesis of ligands, such as glutathione (GSH) (γ-Glu-Cys-Gly), MTs, PCs, or NA, to avoid uncontrolled interactions with binding sites or precipitation with anions, such as phosphate (Andresen et al., 2018). Consequently, a lower synthesis of Zn ligands leads to higher Zn accumulation in the roots. Zinc was much more accumulated in the roots than the shoots in *Andropogon gayanus* exposed to 142.3 up to 854.0 μmol Zn L^–1^ for 7 weeks (Ribeiro et al., 2020). Nardis et al. (2018) mentioned that in different genotypes of *P. maximum*, the Zn concentrations in the roots were higher than in the shoots. On the other hand, these authors also pointed out that in *B. brizantha* cv. Xaraes and *B. decumbens*, the Zn concentration was higher in the stem base (sometimes referred to as the basal node), followed by the shoot and roots, respectively. Commonly, the nodes of grasses contain concentrations of Zn that are more than ten times higher than those of other tissues at both the vegetative and reproductive stages. Grasses use the nodes as hubs for Zn distribution (Yamaji and Ma, 2014). Also, other factors influence Zn accumulation in grasses. Deng et al. (2006) investigated Zn accumulation in *Beckmannia syzigachne*, *Leersia hexandra*, *Neyraudia reynaudiana*, and *Polypogon fugax*, and found that the main tissue with the highest Zn concentration (roots or shoots) greatly varied in function of the Zn concentration in the growth medium, the plant species and the tolerance mechanisms employed to avoid Zn toxicity.

Zinc toxicity restricts both cell division and cell elongation in the roots, impairs cortical root cells, and inhibits Fe translocation (Rout and Das, 2003). Root elongation in *B. syzigachne*, *L. hexandra*, *N. reynaudiana*, and *P. fugax* decreased due to Zn excess (Deng et al., 2006). In *P. maximum*, *B. brizantha*, and *B. decumbens*, Zn toxicity also led to lower root growth (Nardis et al., 2018). In *A. gayanus*, Zn toxicity induced the vesiculation, vacuolation, and withdrawal of the plasma membrane from the root cell wall (Ribeiro et al., 2020). Zinc can also affect proteins, mainly the –SH groups of the plasma membrane and affects membrane stability. In addition, excess Zn can inhibit photosynthesis by affecting both photochemical processes and gas exchange (Rout and Das, 2003). Toxicity symptoms often become visible at leaf Zn concentrations higher than 300 mg kg^–1^ DW, but the toxicity thresholds can be highly variable even within the same species (Broadley et al., 2007). *Lolium perenne* contained more than 500 mg Zn kg^–1^ in its leaves when exposed to 1 mmol Zn L^–1^ or higher for 8 days, which decreased effective quantum yield of PSII (ϕPSII), maximal PSII photochemical efficiency (F_*v*_/F_*m*_), chlorophyll concentration, net photosynthetic rate, stomatal conductance, tillering and biomass yield (Monnet et al., 2005). Zinc can also change the normal functioning of the ascorbate-glutathione (AsA-GSH) cycle, affecting ROS scavenging in grasses (Rabêlo and Borgo, 2016). More symptoms of Zn-induced toxicity in grasses are presented in Table 1.

## Tolerance Mechanisms of Grasses to Trace Element Toxicity

Trace element tolerance is not a simple physiological attribute, but a syndrome of adaptations at the cellular and biochemical levels (Baker, 1981). These adaptations appear to be involved primarily in avoiding the build-up of toxic concentrations at sensitive sites within the cells to prevent damaging effects (Hall, 2002). The strategies involved are diverse: (i) extracellularly they include the immobilization of trace elements by mycorrhizal associations, binding to cell walls, and chelation by root exudates; (ii) tolerance can also involve the plasma membrane, either by reducing the uptake of trace elements or by stimulating the efflux pumping of trace elements that have entered the cytosol; and (iii) within the cytosol, a variety of potential mechanisms exist, for example, for the repair of stress-damaged proteins involving heat shock proteins or MTs, and for the chelation of trace elements by organic acids, amino acids or peptides, or their compartmentation away from metabolic processes by transport into the vacuole (Hall, 2002; Kushwaha et al., 2016). When the employment of these tolerance mechanisms by plants is not sufficient to decrease the fraction of free trace elements within the cell, the antioxidative defense machinery is the next important ‘line of defense’ against trace element-induced toxicity (Gratão et al., 2005). Mechanisms that operate at the whole plant level, such as root-to-shoot transport, have an important role in trace element tolerance, but they are not covered in this section since they were briefly addressed above for each trace element. Specific tolerance mechanisms such as the release of Hg^0^ through the leaves to reduce Hg toxicity (Windham et al., 2001) were also not covered in this section.

### The Role of Mycorrhizas in (Limiting) Trace Element Uptake

Mycorrhizal associations provide an effective exclusion barrier against trace element uptake by adopting absorption, adsorption, or chelation mechanisms that restrict the entry of trace elements into the host plant (Hall, 2002). The excess of Cr, Cu, and Ni was sequestered into the vesicles of arbuscular mycorrhizas (AM) living in the roots of *C. dactylon* grown in gold (Au) and uranium (U) mine tailings, which decreased trace element availability and toxicity to the host (Weiersbye et al., 1999). Inoculation of *B. decumbens* with a mix of AM fungi (*Acaulospora morrowiae*, *Gigaspora albida*, and *Glomus clarum*) inhibited Cd, Cu, and Zn translocation from the roots to the shoots by retaining these elements at the level of the roots and maintaining the trace element concentrations at below toxicity-critical concentration (Soares and Siqueira, 2008). Similarly, the inoculation of *C. dactylon* with two AM fungi (*Funneliformis mosseae* and *Septoglomus constrictum*) decreased both the Pb TF and the Pb bioconcentration factor (BCF) (Mahohi and Raiesi, 2021). Nonetheless, the beneficial effects of AM fungi on *C. dactylon* growth can be affected by the strong antagonistic interaction between AM fungi and plant growth-promoting rhizobacteria in polluted soils in the vicinity of Pb-mining sites (Mahohi and Raiesi, 2021).

The mechanisms of mycorrhizal associations that confer increased trace element tolerance to their host plants have proven to be difficult to resolve and may be diverse and present considerable plant species and trace element specificity since large differences in response to trace elements have been found between fungal species and to different trace elements within species (Hall, 2002). For instance, AM fungi colonization strongly correlated with the species *A. capillaris*, *Arrhenatherum elatius*, and *Calamagrostis epigeios*, and poorly correlated with the chemical characteristics of soil polluted with As and Pb (Teodoro et al., 2020). Although mycorrhizal associations can enhance plant tolerance to trace elements, studies about it in grasses are scarce, being an important gap to be closed in the future.

### The Effect of Root Exudates on Trace Element Availability and Uptake

Plants secrete numerous metabolites from their roots into the rhizosphere to change the pH or to form trace element-metabolite complexes to manage nutrient bioavailability and cope with environmental trace element stresses (Chen et al., 2017). These exudates are a complex mixture of inorganic ions, gaseous molecules [e.g., carbon dioxide (CO_2_), hydrogen gas (H_2_)], and mainly carbon-based compounds that can be subdivided into low-molecular-weight compounds including amino acids, organic acids, sugars, and phenolics, and high-molecular-weight compounds that include mucilage and proteins (Kushwaha et al., 2016; Chen et al., 2017). Malinowski et al. (2004) aimed to identify the effects of endophytic bacterial strains and phosphorus (P) nutrition on Cu acquisition by *F. arundinacea* and observed that Cu^2+^-binding activity by the root exudates of this grass was not affected by endophyte infection but was higher (i.e., lower concentration of free Cu^2+^) in the absence of P.

The accumulation of Cd, Cu, and Pb by *Echinochloa crus-galli* grown in soils polluted with 600 mg Pb kg^–1^ soil, 40 mg Cd kg^–1^ soil, and 100 mg Cu kg^–1^ soil was 2- to 4-fold higher when this grass was assisted with root exudates from *Belamcanda chinensis* for 4 weeks (Kim et al., 2010). The root exudates also increased the BCF and TF for Cd, Cu, and Pb, which indicates that root exudates can have an important role in phytoextraction efficiency. However, the Cd bioavailability decreased due to malate exudation by *S. bicolor* exposed to Cd (0, 0.5, and 5 mg L^–1^ in solution) (Pinto et al., 2008), supporting the idea that the trace element-binding capabilities of root exudates can be important for trace element phytostabilization. The secretion types and patterns of root exudates vary in the function of plant species, physiological conditions, and soil environment (Chen et al., 2017), and apparently, in the function of these conditions, root exudates can contribute to phytoextraction (mainly of sites with a low level of trace elements pollution) or phytostabilization (Pinto et al., 2008; Kim et al., 2010). Indeed, trace element uptake can be restricted by immobilizing trace elements, limiting their passage through the plasma membrane (Hall, 2002), or favored by the formation of chelates, facilitating uptake in the cell and translocation of trace elements from the roots to shoots (Kushwaha et al., 2016). Nonetheless, studies about the role of root exudates in trace element tolerance in grasses considered for phytoextraction or phytostabilization are largely lacking. Most of the existing studies have focussed on the role of root exudates in Fe and Zn uptake by grasses in the context of plant mineral nutrition (Chen et al., 2017). Cakmak et al. (1996), for instance, reported that the release of phytosiderophores by Zn deficient *A. orientale* increased Zn uptake.

### The Functions of Cell Walls in Tolerance Mechanisms

Once trace elements have crossed the barrier provided by mycorrhiza or have not been chelated by root exudates, they face another possible ‘line of defense’ against trace element-induced toxicity. Cell walls tend to bind divalent and trivalent trace element cations during their uptake by cells and at the final stage of their sequestration from the cytosol to decrease the toxicity due to free trace element into the cells (Hall, 2002; Krzesłowska, 2011). Almost 60% of Cd retained in the roots of *P. maximum* and *B. decumbens* was situated in the root apoplast, probably adsorbed to the cell walls (Rabêlo et al., 2021). Similarly, in *P. australis*, most Zn accumulated within the root apoplast and Zn concentrations followed the gradient: intercellular spaces > cell walls > vacuoles > cytosol (Jiang and Wang, 2008). Most of the Pb absorbed by *B. decumbens* was present in the root apoplast as chloropyromorphite, which thickened the cell walls surrounding these crystalline Pb deposits, indicating that the sequestration of insoluble Pb into the cell walls represents an important mechanism of Pb tolerance (Kopittke et al., 2008). Divalent (e.g., Cd^2+^, Pb^2+^, and Zn^2+^) and trivalent trace element cations can bind to functional groups of the cell wall, such as carboxyl (–COOH), hydroxyl (–OH), and –SH (Kushwaha et al., 2016).

Both, the primary and secondary cell walls have a range of mechanisms that can be customized to cope with trace elements. Pectins contain most of the negative charges within the primary cell wall and can sequester trace elements efficiently (Krzesłowska, 2011; Loix et al., 2017). Binding studies with low-methylesterified pectins showed that the affinity of divalent and trivalent trace element cations depends on the origin of pectins. Among the bivalent trace element cations, Cu^2+^ and Pb^2+^ are more strongly bound to pectins, whilst the binding of Zn^2+^ and Ni^2+^ is less strong (Krzesłowska, 2011). However, the type II of the primary wall and secondary wall, characteristic mainly for grasses, normally contain much fewer pectins (Pelloux et al., 2007). Pectins had no significant contribution to Cu binding in the cell wall of *Paspalum notatum* (Nabais et al., 2011). However, Ni was substantially bound to pectins in young roots (21 days), but not in older roots (120 days) (Nabais et al., 2011), probably due to a higher proportion of secondary cell walls in the older roots. The incrustation of phenolic components such as lignin during the formation of the secondary cell wall is an important process for the immobilization of trace elements and to create a stronger barrier for their entry (Krzesłowska, 2011; Loix et al., 2017). The amount of the cell wall-bound phenolics in *V. zizanioides* increased due to Cr, Cu, Ni, Pb, or Zn exposure compared with the non-exposed control conditions. This was different from what occurred after As exposure which caused the most severe phytotoxicity in this species (Melato et al., 2012). The genes involved in the lignin synthesis were upregulated due to Cd exposure in both the roots and shoots of *P. americanum* × *purpureum* (Zhao J. et al., 2019). An increased lignification and thickening of the cell layers of endodermis and exodermis in the root tissues and the cell walls of the xylem and cortical parenchyma was reported in *B. decumbens* exposed to Cd, Cu, Pb, and Zn (Gomes et al., 2011) and *P. maximum* exposed to Cd (Rabêlo et al., 2018c). Changes in the cell wall composition contribute to the trace element tolerance in both excluder and accumulator species, but the affinity of the trace element to the root cell wall tends to be different between them. Trace elements bound to the root cell wall of accumulators remain more available for xylem loading than in excluders or non-accumulator plants (Li et al., 2007).

### The Role of Plasma Membranes and Trace Element Efflux

The role of the plasma membrane in trace element tolerance can either be by reducing the uptake of trace elements or by the increased efflux pumping of the trace elements that entered the cytosol (Hall, 2002). In this section, only the role of the plasma membrane in trace element efflux is addressed. After being taken up in the cells, a trace element can either be returned to the soil solution or remain in the apoplast. The direction of trace element efflux corresponds to the trace element accumulation phenotype of the plant. For instance, in non-accumulator plants, root efflux transporters direct trace elements to the soil solution, whilst in accumulators, the efflux system is directed toward loading trace elements into the xylem, on their way to the shoots (Lin and Aarts, 2012). With the objective to decrease As toxicity, *P. clandestinum* exposed to AsO_4_^3^**^–^** (1, 5, 10, and 20 μmol L**^–^**^1^) reduced the AsO_4_^3^**^–^** into AsO_3_^3^**^–^** and realized a fast AsO_3_^3^**^–^** efflux from the roots to the nutrient solution (Panuccio et al., 2012). Likely, *P. clandestinum* is a non-accumulator species for As. Although a fast AsO_3_^3^**^–^** efflux was observed, no transporters involved in As efflux were reported. Some transporters can act in trace element efflux, such as transporters of the P_1*B*_-ATPase and ATP-binding cassette (ABC) families (Lin and Aarts, 2012), but information about their role in grasses is not available. The expression of *ABC* genes in the roots and shoots of *F. arundinacea* exposed to Ni was reported to be higher compared with the control (Shabani et al., 2016), but no more details were provided.

### Chelation of Trace Elements by Ligands and Transport Into Vacuoles

Organic acids, amino acids, and (poly)peptides are potential ligands for trace elements and thus, can play a role in trace element tolerance and detoxification (Cobbett and Goldsbrough, 2002; Sharma and Dietz, 2006; Osmolovskaya et al., 2018). The role of organic acids in the intracellular detoxification of trace elements has been studied most intensively in hyperaccumulators, especially in Zn hyperaccumulator species (Osmolovskaya et al., 2018). In such plants, Zn is chelated by malate to form a relatively weak complex in the cytosol that is transported into the vacuoles, where the complex dissociates and Zn^2+^ binds with stronger chelators such as citrate or oxalate. Malate is re-exported to the cytosol (Osmolovskaya et al., 2018). The involvement of malate, citrate, and oxalate in trace element tolerance was also demonstrated in non-hyperaccumulators such as *L. perenne*, which presented increased concentrations of these organic acids in the shoot under Ni exposure (Yang et al., 1997). Oxalate may participate in Cd sequestration into the vacuole in roots and also in Cd translocation to the shoot in *S. alternifloria* (Chai et al., 2012). On the other hand, the concentrations of organic acids such as malate, citrate, and fumarate were not higher in the roots, stems, or leaves of Cd-exposed *P. maximum* than in non-exposed ones (Rabêlo et al., 2018b). Probably other mechanisms of tolerance to Cd such as enhanced synthesis of amino acids, GSH, and PCs are more important than organic acids for Cd detoxification in *P. maximum* (Rabêlo et al., 2019).

Upon exposure to toxic concentrations of trace elements, plants often synthesize a range of diverse metabolites that accumulate to concentrations in the millimolar range, particularly specific amino acids, such as proline and histidine (Sharma and Dietz, 2006). An increased accumulation of proline was described in the leaves of *P. maximum* exposed to Cu (Gilabel et al., 2014) and *C. dactylon* exposed to Cd (Xie et al., 2014). The proline-inducing ability of different trace elements varies, and when compared at equimolar concentrations in the growth medium, Cu is the most effective in inducing proline accumulation followed by Cd and Zn, respectively (Schat et al., 1997). Proline functions as an osmolyte, radical scavenger, electron sink, stabilizer of macromolecules, and a cell wall component (Sharma and Dietz, 2006). Trace element-tolerant plants, like Zn-tolerant ecotypes of *Deschampsia cespitosa*, possess substantially elevated constitutive proline levels in different plant parts (Smirnoff and Stewart, 1987). Some studies also reported increased histidine concentrations induced by toxic concentrations of trace elements. Nickel exposure led to higher histidine concentrations in the roots of *D. cespitosa* (Nguyen et al., 2018), while histidine concentrations increased in the roots, stems, and leaves of *P. maximum* exposed to Cd (Rabêlo et al., 2018b). Histidine can also bind other divalent ions such as Cu and Zn, decreasing their toxicity and the level of induction of oxidative stress in the cytosol (Zheng et al., 2013). However, the chelation of trace elements (e.g., Ni) by free histidine accumulated in the roots inhibits the trace element sequestration into the vacuole and promotes trace element translocation from the roots to shoots (Richau et al., 2009). Besides proline and histidine, other amino acids such as cysteine can be more accumulated under trace element exposure in grasses (Rabêlo et al., 2017b), probably due to their use for peptides synthesis.

Phytochelatins and MTs are the best characterized and probably the most important trace element-binding ligands in plant cells (Cobbett and Goldsbrough, 2002). Phytochelatins act on trace element chelation and are involved in their transport from the cytosol to the vacuole and also in its translocation from the roots to shoots (Cobbett and Goldsbrough, 2002; Mendoza-Cózatl et al., 2008). In the roots and stems of *P. maximum*, the synthesis of PCs was strongly increased by Cd exposure (Rabêlo et al., 2018b). Phytochelatins are synthesized inductively by exposure to not only Cd but also by other trace elements such as Costal, Cu, Hg, Pb, and Zn (Yadav, 2010), as reported in the roots of *L. perenne* exposed to As (Schulz et al., 2008) and *V. zizanioides* exposed to Pb (Andra et al., 2010). The processes involved in the transport of trace element-PCs complexes from the cytosol to the vacuole were reviewed in detail (Sharma et al., 2016). Although PCs are important players in trace element detoxification, they are not always induced under trace element exposure due to the combined action of other detoxification processes (Rabêlo et al., 2019), as observed in *D. cespitosa* exposed to Ni (Hayward et al., 2013) and *B. decumbens* and *P. maximum* exposed to Cd (Rabêlo et al., 2021). Besides the PCs, MTs are supposed to function as trace element-binding ligands in plant cells (Cobbett and Goldsbrough, 2002), although their role in plants remains to be confirmed. They could play a role in trace element metabolism, but their precise function is not clear; they may have distinct functions for different trace elements; alternatively, they could function as antioxidants, although evidence is lacking, while a role in plasma membrane repair is another option (Hall, 2002). The expression of *MET* genes that encode for MTs increased in the roots and shoots of *F. arundinacea* exposed to 30 mg Ni kg^–1^ soil compared with non-exposed plants (Shabani et al., 2016). Ukoh et al. (2019) observed that the MTs concentration increased in the roots and stems of *P. maximum* exposed to 5 mg Zn kg^–1^ soil compared with the non-exposed controls, but there was no MTs increase in the leaves.

Trace elements not bound to chelators also can be transported to vacuoles through a series of transporters (Sharma et al., 2016). However, studies about this are scarce in grasses. To the best of our knowledge, only two studies showing the role of the vacuolar Na^+^/H^+^ antiporter (*NHX1*) and the expression of *HMA3*, a P_1*B*_-ATPase, in Cd transport from cytosol to vacuoles in *Leptochloa fusca* and *Festulolium loliaceum*, respectively, are currently available (Adabnejad et al., 2015; Guo et al., 2017).

### Antioxidative Machinery in Relation to Trace Element Toxicity

Oxidative stress is considered as a primary effect of the exposure to toxic concentrations of trace elements (e.g., Cd) (Clemens, 2006**;** Cuypers et al., 2016). This scenario occurs when there is a serious imbalance in any cell compartment between the production of ROS (e.g., O_2_^⋅–^, H_2_O_2_, and ^⋅^OH) and antioxidative defense. This can be initiated due to the insufficient efficiency of the tolerance mechanisms described above (Gratão et al., 2005). According to Hall (2002), trace element-tolerant plants do not appear to possess enhanced tolerance to free radicals or ROS, but rather, rely on improved mechanisms for trace element homeostasis. For instance, enzymes involved in ROS scavenging [e.g., superoxide dismutase (SOD) (EC 1.15.1.1), catalase (CAT) (EC 1.11.1.6), and ascorbate peroxidase (APX) (EC 1.11.1.11)] are not principal in the oxidative stress control in the roots, stems, and leaves of *P. maximum* that is considered as a Cd-tolerant non-hyperaccumulator species, since it can promote the vacuolar sequestration of Cd from the cytosol (Rabêlo et al., 2018b,d). This suggests that plants that are not equipped with a trace elements tolerance mechanism need a higher antioxidative capacity (non-enzymatic and enzymatic) to cope with trace element-induced stress than trace element-tolerant plants. Several non-enzymatic antioxidants such as ascorbate, GSH, flavonoids, alkaloids, tocopherols, carotenoids, phenolic compounds, and non-protein amino acids are employed by plants for ROS scavenging (Gratão et al., 2005). Among them, GSH, which can be oxidized to oxidized glutathione (GSSG) avoiding oxidative damages in sensitive organelles, is considered the principal non-enzymatic antioxidant in plants (Yadav, 2010; Seth et al., 2012). Moreover, GSH is used as a substrate for PCs synthesis and can also act as a ligand for trace elements, besides transmitting specific information tuning cellular signaling pathways under trace element stress conditions (for a comprehensive review see Seth et al., 2012). The beneficial effects of GSH on trace element tolerance were described in *L. perenne* grown in soil polluted with As, Cu, Hg, Pb, and Zn (Anjum et al., 2013), *Poa pratensis* exposed to Cu (Liu et al., 2016), and both *P. maximum* and *B. decumbens* exposed to Cd (Rabêlo et al., 2021). Under high Cd accumulation, symptoms of oxidative stress, such as lipid peroxidation, can occur as a consequence of GSH depletion due to the binding of Cd^2+^ to GSH and the formation of GSH-derived PCs (Clemens, 2006). Sugars such as galactinol and raffinose that exhibit a similar capacity as GSH to scavenge ^⋅^OH radicals (Nishizawa et al., 2008) were also shown to be important in oxidative stress control in grasses like *P. maximum* exposed to Cd (Rabêlo et al., 2018b,d). The major ROS-scavenging mechanisms of plants include enzymes of the AsA-GSH cycle as SOD that dismutates O_2_^⋅–^ to H_2_O_2_, and CAT, APX, and guaiacol peroxidase (GPX) (EC 1.11.1.7) that are involved in reducing H_2_O_2_ into H_2_O (Gratão et al., 2005). Ullah et al. (2020a) reported that, after 90 days of growth, the SOD activity was enhanced in the leaves of *B. mutica* and *L. fusca* exposed to Cd (10 and 50 mg Cd kg**^–^**^1^ soil) or Pb (50 and 250 mg Pb kg**^–^**^1^ soil) compared with the non-exposed plants. Similarly, the SOD activity increased in both the roots and shoot of *V. zizanioides* exposed to AsO_4_^3^**^–^** or AsO_3_^3^**^–^** (0, 25, 50, 100, 200, 400, and 800 μmol L**^–^**^1^) for 28 days (Yu et al., 2020). In contrast, the activities of Mn-SOD and Cu/Zn-SOD did not increase in the roots, stems, and leaves of *P. maximum* exposed to Cd (100 μmol L**^–^**^1^) for 9 days (Rabêlo et al., 2018d).

**FIGURE 4:**
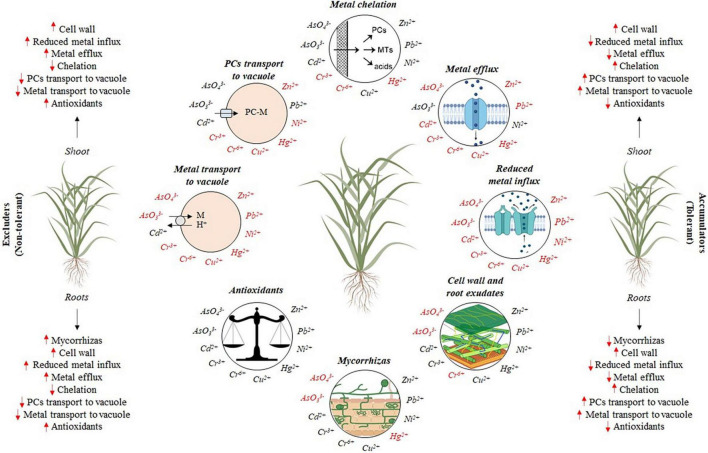
Summary of cellular mechanisms involved in metal detoxification and tolerance in grasses: (i) restriction of metal movement to roots by mycorrhizas; (ii) binding to the cell wall and root exudates; (iii) reduced influx across plasma membrane; (iv) active efflux into the apoplast; (v) chelation in the cytosol by phytochelatins (PCs), metallothioneins (MTs), amino acids and organic acids; (vi) transport of PC-M complex into the vacuoles; (vii) transport and accumulation of metal (M) not chelated into the vacuole; (viii) the balance between antioxidants and oxidants. Adapted from Hall (2002). Red arrows indicate which tolerance mechanisms are possibly more (↑) or less (↓) requested by excluder and accumulator grasses. Metals written in red indicate the need for further studies in relation to each specific tolerance mechanism or indicate that the tolerance mechanism is not employed in its detoxification.

The activity of the enzymes of the AsA-GSH cycle is quite variable in grasses and depends on the concentration of the trace elements, exposure time, environmental conditions, plant nutritional status, and the action of other tolerance mechanisms (Rabêlo and Borgo, 2016; Rabêlo et al., 2018a). The activity of APX in the leaves of *P. pratensis* increased due to Cu exposure (Liu et al., 2016), as well as the glutathione reductase activity (GR) (EC 1.6.4.2), which reduces GSSG into GSH; monodehydroascorbate reductase (MDHAR) (EC 1.6.5.4) and dehydroascorbate reductase (DHAR) (EC 1.8.5.1) that act on AsA recycling (Gratão et al., 2005). However, the activities of CAT, GPX, and GR did not enhance in the *L. perenne* simultaneously exposed to As, Cu, Hg, Pb, and Zn (Anjum et al., 2013). These findings indicate that specific aspects related to plant species and tissues must be considered to interpret antioxidative responses under trace element exposure. A simplified and general summary of the potential cellular mechanisms involved in trace element detoxification and tolerance is presented in Figure 4 for both excluder and accumulator grasses used for trace elements phytoremediation.

## Use of Grasses for Trace Element Phytoremediation

Plants can be divided into four groups with respect to their responses for uptake and tolerance to trace elements: (i) “regular” plants only tolerate low concentrations of available trace elements in the soil before they die due to phytotoxicity; (ii) excluders can grow over a wide range of available phytotoxic trace elements before physiological mechanisms lose control and allow for unregulated uptake, resulting in the death of the plant; (iii) bioindicators take up trace elements over a wider range than “regular” plants and the concentrations in the plant leaves reflect those of the soil until phytotoxicity prevents further growth and causes the death of the plant; and (iv) (hyper)accumulators can tolerate much higher concentrations of bioavailable trace elements than “regular” plants, bioindicators, and excluders (Baker, 1981; van der Ent et al., 2013). The most interesting groups for phytoremediation purposes are excluders, which normally accumulate trace elements in the roots withy very limited translocation to the shoots (Awa and Hadibarata, 2020) and are recommended for phytostabilization (Alkorta et al., 2010), and (hyper)accumulators, which preferentially accumulate trace elements in the shoot (Awa and Hadibarata, 2020) and can be used for phytoextraction (Vangronsveld et al., 2009). Bioindicators with a high biomass yield can be used also for phytoextraction in moderately polluted agricultural soils (Rabêlo et al., 2020a). Most grass species are trace element excluders (Fernández et al., 2017), but there exist many bioindicators and a few accumulators as well. Both excluders and accumulators tolerant to hypoxia can also be used to remove trace elements from polluted wastewaters, groundwaters, and surface waters; this approach is known as rhizofiltration or phytofiltration (Awa and Hadibarata, 2020). In order to screen grasses according to their use for trace element phytoremediation, we briefly described below the main aspects associated with phytoextraction, phytostabilization, and phytofiltration, and examples of appropriate species for each strategy, considering the fact that there exists trace element specificity within most grass species. Therefore, the same species could be a suitable candidate for the phytoextraction of trace element *x* and phytostabilization of trace element *y*, or in some cases (e.g., *A. capillaris*) could be a suitable candidate for both phytoextraction and phytostabilization of the same trace element depending on the composition of its rhizosphere microbiome (Truyens et al., 2014).

### Phytoextraction

It is necessary to consider that there exist important limitations for trace element phytoextraction: (i) it can only be applied to low to moderately polluted soils, and (ii) its applicability depends on the depth of rooting, which varies with the plant species used, the soil type, and the depth of the groundwater table, but on average is not more than 50 cm (Vangronsveld et al., 2009). An ideal plant for trace element phytoextraction should possess the following characteristics: (i) tolerant to the trace element accumulated, (ii) fast-growing and high biomass, (iii) accumulating trace elements in the above-ground parts, and (iv) easy to harvest (Vangronsveld et al., 2009). Furthermore, another point requiring caution when choosing plant species for phytoextraction purposes is that a non-specific “breakthrough” of trace element uptake into the shoots may occur when the tolerance limit of an excluder species is exceeded (Baker, 1981). However, this does not imply that such species can be considered as an (hyper)accumulator if the high concentration of the trace element absorbed results in the death of the plant (van der Ent et al., 2013). Most (hyper)accumulators do not occur on non-metal-enriched soils due to the competitive disadvantages and greater sensitivity to fungal and pathogen infections (van der Ent et al., 2013) unlike most of the grasses listed as suitable choices for trace element phytoextraction in Table 2. Given that most, grass species also occur on “normal” soils, they are pseudometallophytes (Tordoff et al., 2000). Probably, most grass species that have been assessed for trace element phytoextraction are actually bioindicator plants, for example *B. decumbens* and *P. maximum* (Rabêlo et al., 2020a). Thus, the use of such plants for trace element phytoextraction is feasible only in mildly polluted agricultural soils, otherwise trace element-induced phytotoxicity will prevent further growth and biomass yield. In the latter case, the use of grasses used for trace element phytoextraction will be very inefficient, especially if compared to hyperaccumulators.

**TABLE 2:** Examples of suitable grass species for each strategy of phytoremediation in sites contaminated with potentially toxic trace elements.

Grass species	Trace elements	References
**Phytoextraction**		
*Agrostis capillaris*	Cd	Truyens et al., 2014
*Agrostis delicatula*	As, Cu, Pb, Zn	Gomes et al., 2014
*Brachiaria decumbens^1^*	Cd, Pb, Zn	Santos et al., 2006; Rabêlo et al., 2020a
*Brachiaria dictyoneura*	Cd, Hg	Arroyave et al., 2010
*Brachiaria mutica^2^*	Cd, Pb	Ullah et al., 2019
*Bromus squarrosus*	Cd, Zn	Hesami et al., 2018
*Cynodon dactylon*	Cd, Cu, Ni, Zn	Chandra et al., 2018; Opoku et al., 2020; Gajaje et al., 2021
*Desmostachya bipinnata*	Cd	Ullah et al., 2019
*Eremochloa ciliaris*	Hg	Qian et al., 2018
*Festuca rubra*	Cr	Lago-Vila et al., 2015
*Imperata cylindrical*	Hg	Fu et al., 2016
*Leptochloa fusca*	Cd	Ullah et al., 2019
*Lolium perenne*	Cd, Pb, Zn	Zhang J. et al., 2019; Zhang Y. et al., 2019
*Lolium temulentum*	Cd	Marzban et al., 2017
*Miscanthus* × *giganteus*	Cr, Ni	Stypczyńska et al., 2018
*Miscanthus sacchariflorus*	Cr, Ni, Zn	Li et al., 2014; Stypczyńska et al., 2018
*Miscanthus sinensis*	Hg	Zhao A. et al., 2019
*Panicum maximum^3^*	Cd, Cu	Azeez et al., 2020; Rabêlo et al., 2020a
*Paspalum fasciculatum*	Pb	Salas-Moreno and Marrugo-Negrete, 2020
*Paspalum viginatum*	Cd	Opoku et al., 2020
*Pennisetum purpureum^4^*	Cd, Cu, Ni, Zn	Chandra et al., 2018; Hou et al., 2020
*Poa bulbosa*	Zn	Hesami et al., 2018
*Saccharum munja*	Cu, Ni, Zn	Chandra et al., 2018
*Spartina argentinensis*	Cr	Redondo-Gómez et al., 2011
*Sporobolus arabicus*	Cd	Ullah et al., 2019
*Vetiveria zizanioides^5^*	Cd, Pb, Zn	Antiochia et al., 2007; Opoku et al., 2020
**Phytostabilization**		
*Agrostis capillaris*	As, Cd, Pb, Zn	Teodoro et al., 2020
*Agrostis castellana*	As, Zn	Freitas et al., 2009
*Agrostis durieui*	Pb	Fernández et al., 2017
*Arrhenatherum elatius*	As, Cd, Pb, Zn	Teodoro et al., 2020
*Avena sativa*	Cr, Cu, Hg, Pb, Zn	Fitamo et al., 2011; Gutiérrez-Ginés et al., 2012; Amin et al., 2019
*Avena strigosa*	Cd, Cr, Pb	Hoehne et al., 2016
*Brachiaria decumbens*	Pb	Andrade et al., 2014
*Brachiaria mutica*	Cr	Kullu et al., 2020
*Brachiaria raptans*	Cu, Pb	Nazir et al., 2011
*Bromus inermis*	As, Pb, Zn	Turnau et al., 2010
*Bromus tectorum*	Ni, Zn	Sinegani and Dastjerdi, 2009
*Bromus tomentellus*	Cr, Zn	Roohi et al., 2020
*Bromus tomentellus*	As	Sharifi et al., 2012
*Calamagrostis epigeios*	As, Cd, Pb, Zn	Teodoro et al., 2020
*Chloris gayana*	Cd, Cu	Fitamo et al., 2011
*Chloris virgata*	Cd, Cr, Pb	Mishra et al., 2020
*Cynodon dactylon*	Cd, Cr, Pb, Zn	Yang et al., 2014; Mishra et al., 2020
*Dactylis glomerata*	As, Cd, Pb, Zn	Visconti et al., 2020
*Desmostachya bipinnata*	Cd, Cr, Pb	Mishra et al., 2020
*Digitaria sanguinalis*	Cd, Pb, Zn	Yang et al., 2014; Wang et al., 2020
*Elymus elongatus^6^*	Cd, Ni	Boros-Lajszner et al., 2021
*Elymus hispidus*	As, Pb, Zn	Turnau et al., 2010
*Elymus macrourus*	Cu, Pb, Zn	Busby et al., 2020
*Eremochloa ophiuroides*	Cd	Liu et al., 2012
*Festuca arundinacea^7^*	Cd, Ni, Pb, Zn	Albornoz et al., 2016; Gómez et al., 2016; Steliga and Kluk, 2020
*Festuca rubra*	As, Cd, Hg, Ni, Zn	Ebbs et al., 1997; Lago-Vila et al., 2015; Fernández et al., 2017
*Imperata cylindrical*	Cd, Cu, Pb, Zn	Peng et al., 2006
*Leptochloa fusca*	Cr	Ullah et al., 2021
*Lolium perenne*	Cd, Cu, Ni, Pb	Radziemska et al., 2017
*Lygeum spartum*	Cu, Pb, Zn	Conesa and Faz, 2011
*Melica transsilvanica*	As, Pb, Zn	Turnau et al., 2010
*Miscanthus* × *giganteus*	Cd, Cu, Hg, Pb, Zn	Kocoń and Jurga, 2017; Zgorelec et al., 2020
*Miscanthus floridulus*	Cd, Cu, Pb, Zn	Peng et al., 2006
*Miscanthus sinensis*	Cd	Hou et al., 2020
*Panicum virgatum*	Cd, Zn	Rusinowski et al., 2019; Hou et al., 2020
*Paspalum fasciculatum*	Cd	Salas-Moreno and Marrugo-Negrete, 2020
*Paspalum plicatulum*	Cu	De Conti et al., 2020
*Pennisetum americanum* × *purpureum*	Cd, Cu, Pb, Zn	Zhang et al., 2016
*Pennisetum purpureum*	Zn	Hou et al., 2020
*Pennisetum purpureum* × *thyphoideum*	As, Cd, Pb, Zn	Gao et al., 2020
*Phragmites australis*	As	Sharifi et al., 2012
*Piptatherum miliaceum*	Cu, Pb, Zn	Conesa and Faz, 2011
*Rendlia altera*	Cu	Shutcha et al., 2010
*Setaria sphacelata*	Cd, Cu, Zn	Fitamo et al., 2011
*Spartina pectinata*	Cd, Zn	Korzeniowska and Stanislawska-Glubiak, 2015; Rusinowski et al., 2019
*Stipagrostis plumosa*	Ni	Tavili et al., 2019
**Phytofiltration**		
*Brachiaria mutica*	Cd, Pb	Khan et al., 2018; Ullah et al., 2020b
*Miscanthus sacchariflorus*	Cd, Cu, Pb, Zn	Yao et al., 2018
*Panicum aquanticum*	Pb	Pires-Lira et al., 2020
*Panicum coloratum*	Hg	Mbanga et al., 2019
*Panicum repens*	Cu, Pb, Zn	El-Gendy et al., 2017
*Phragmites australis*	Cr, Cu, Hg, Ni, Pb, Zn	Mbanga et al., 2019; Odinga et al., 2019

*^1^Known also as Urochloa decumbens;*

*^2^Known also as Urochloa mutica;*

*^3^Known also as Megathyrsus maximus;*

*^4^Known also as Cenchrus purpureus;*

*^5^Known also as Chrysopogon zizanioides;*

*^6^Known also as Agropyron elongatum;*

*^7^Known also as Schedonorus arundinaceus.*

**FIGURE 5:**
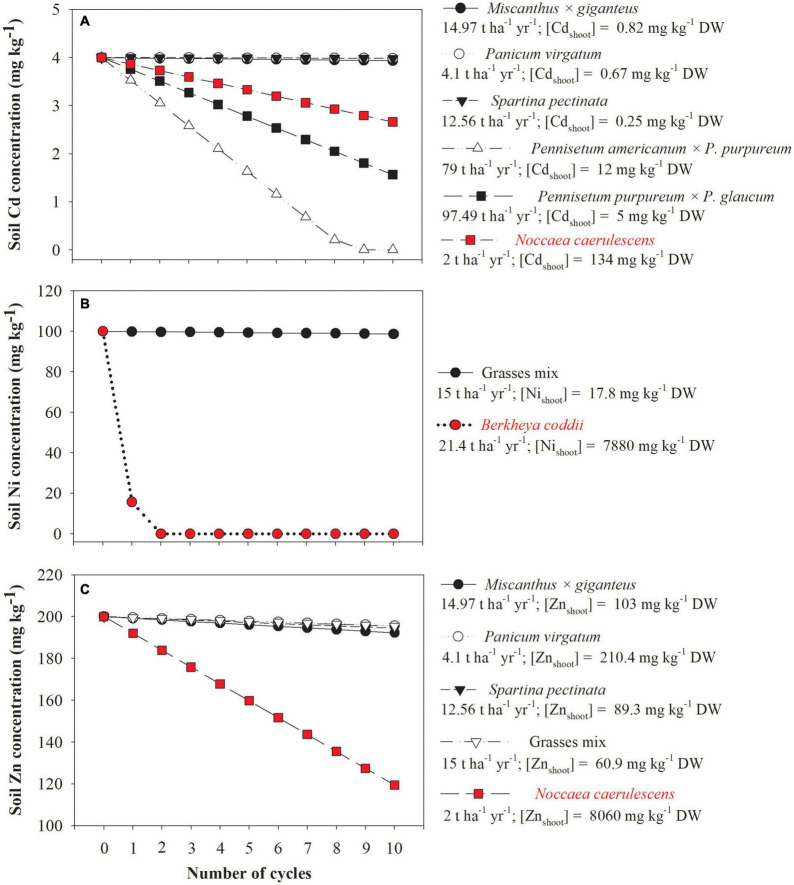
Theoretical reduction on cadmium (Cd, **A**), nickel (Ni, **B**), and zinc (Zn, **C**) concentrations by the cultivation of grasses and known hyperaccumulator plants (highlighted in red color), in a mildly contaminated soil, according to the number of cycles of cultivation (in this specific case, one cycle corresponds to 1 year of cultivation). The assumptions made were: (i) the total soil metal concentration decreases linearly due to a constant yearly extraction, (ii) the contamination and rooting depth are 0.2 m, and (iii) soil density is 1 kg dm^–3^. Grasses mix = *Festuca arundinacea*, *Festuca rubra*, *Lolium perenne*, and *Poa pratensis*. The data of the biomass yield and metals concentration used to plot this figure were extracted from Robinson et al. (1997); Zhang et al. (2014), Kitczak et al. (2016); Jacobs et al. (2018), Rusinowski et al. (2019), and Zhou et al. (2020).

Theoretical calculations indicate that only grasses producing more than 75 t DW ha^–1^ yr^–1^ are as efficient as *Noccaea caerulescens* (a known Cd hyperaccumulator) to reduce Cd concentrations in mildly polluted soils (Figure 5A). Furthermore, the use of grasses producing < 15 t DW ha^–1^ yr^–1^ results in a significant reduction in the concentrations of Cd, Ni, and Zn in polluted soil only after 10 cultivation cycles which differs greatly compared to hyperaccumulators (Figures 5A–C). However, it is necessary to be cautious in interpreting the results of such theoretical calculations because the values used for biomass yield (t DW ha^–1^ yr^–1^) and trace element concentrations were for grasses listed as good candidates for trace elements phytostabilization (Table 2) and suitable data were not found in the same study for phytoextractor grasses. In this respect, the efficiency of grasses listed as good candidates for phytoextraction to reduce trace element concentrations might be closer to a hyperaccumulator, but again, only for mildly polluted soils. In this scenario, some authors have mentioned that effective trace element removal realized by harvesting non-hyperaccumulator plants with high biomass yield is not different from the removal by hyperaccumulators that generally produce less biomass (Ebbs et al., 1997; Kayser et al., 2000). On the other hand, in soils that are more heavily polluted, trace element removal by hyperaccumulator plants is always higher compared with plants with higher biomass (Chaney et al., 1997) due to the phytotoxicity-induced decline of the biomass production of the latter.

Phytoextraction efficiency is often estimated by calculating the BCFs and TFs. It has been suggested that only plant species with both factors > 1 have potential to be used in phytoextraction (Buscaroli, 2017). In this case, the available concentration of the trace elements must be used for calculating the BCF, since the use of (pseudo)total soil concentrations can result in a very distorted representation of the actual viablity especially for trace elements with generally low bioavailability, such as Cr and Pb (Senila, 2014; Ertani et al., 2017). Another point requiring caution to interpret BCFs is that high (total or bioavailable) trace element concentrations in the soil may result in a BCF < 1, but even so, the plant species might be very efficient in sequestering the trace element, but the opposite can also occur (van der Ent et al., 2013). Given this scenario, the use of TF can provide a higher reliability to screen plant species (grasses or not) for their potential phytoextraction capacities since this simple calculation allows for the discrimination between excluder and accumulator plants. Moreover, another point that should be considered when screening the suitability of grass species for phytoextraction is their biomass yield. Besides high BCF and TF, high phytoextraction efficiencies are also directly correlated with high biomass (McGrath and Zhao, 2003), and therefore this parameter should be considered, as shown in Figure 5A.

### Phytostabilization

Phytostabilization is not a technology for the clean-up of polluted soils, but rather a management strategy for stabilizing (or deactivating) elements that are potentially toxic (Vangronsveld et al., 2009). Revegetation stabilizes the polluted soil, establishing a cover that prevents the dispersion of pollutants by water or wind erosion and reduces the mobility and bioavailability of trace elements (Alkorta et al., 2010), thus preventing or restricting their entry into the food chains (Buscaroli, 2017). Plants can immobilize trace elements in soils through sorption by roots, precipitation, complexation, or changing the trace element valence in the rhizosphere (Alkorta et al., 2010). The use of soil amendments (e.g., liming, clays, and zeolites) to support plant growth, including grasses (Gao et al., 2020) can increase phytostabilization efficiency (Vangronsveld et al., 2009).

The choice of plant species is a crucial aspect of trace element phytostabilization. Ideally, plants should develop an extensive root system and large biomass in the presence of high concentrations of trace elements whilst keeping the root-to-shoot translocation as low as possible (Alkorta et al., 2010). In this respect, root colonization by AM fungi can have a great effect on the phytostabilization capacity of grasses such as *A. capillaris*, *A. elatius*, and *C. epigejos* exposed to As, Cd, Pb, or Zn by restricting the entry of trace elements into the host plant (Teodoro et al., 2020). Similarly, the application of soil amendments such as biochar, peat, manure, and even non-polluted soil, has been shown to efficiently keep the BCFs of As, Cd, Pb, and Zn < 1 in the grass *P. purpureum* × *thyphoideum* by decreasing their bioavailability (Gao et al., 2020). Based upon these empirical results, it is clear that assisted phytostabilization is indeed promising, but even so, the plant species selected for trace elements phytostabilization should ideally present both a BCF and a TF < 1 (Buscaroli, 2017). Further details about phytostabilization prospects have been presented by Alkorta et al. (2010). An overview of the most suitable grasses to perform trace element phytostabilization is presented in Table 2.

### Phytofiltration

Phytofiltration employs plant roots to absorb pollutants in the root zone, concentrating and precipitating them onto or within their harvestable root mass (Kristanti et al., 2021). The site of trace elements sorption (on roots, within roots, or in the aerial organs of plants) depends on the type of plant and the speciation and concentration of the specific trace element concerned (Kristanti et al., 2021). Suitable plants selected for trace element phytofiltration should ideally be tolerant to hypoxia and the target trace element(s) and possess an extensive root surface area for sorption (Awa and Hadibarata, 2020). Grasses well adapted to conditions of low dioxygen (O_2_) availability such as *B. mutica* and *P. australis* can be good candidates for trace elements phytofiltration. *Brachiaria mutica* persisted in a hydroponic system containing high Pb concentrations (100, 200, and 500 μmol L^–1^) and retained the major part of Pb ad- and absorbed in its roots (Khan et al., 2018). *Phragmites australis* efficiently decreased the concentrations of Cr, Cu, Ni, Pb, and Zn in a phytofiltration system for wastewaters (Odinga et al., 2019). Terrestrial plants normally have extensive fibrous roots that provide a larger surface area for trace elements sorption than aquatic plants (Awa and Hadibarata, 2020; Kristanti et al., 2021). However, *V. zizanioides* exposed to landfill leachate were shown to accumulate high concentrations of Cr, Ni, and Zn (Fasani et al., 2019). Nonetheless, its growth under leachate exposure was strongly dependent on leachate composition, making a case-by-case evaluation of plant tolerance necessary before any large-scale application. Moreover, the exposure time, ambient temperature, and pH of the water to be treated also have a primary influence on the responses of such plants used for any phytofiltration system (Kristanti et al., 2021). From a broad consideration of the issues mentioned in this section, it emerges that currently, only a few grass species presented satisfactory results for their application in trace element phytofiltration (see Table 2).

## Strategies to Improve the Efficiency of Trace Element Phytoremediation by Grasses

Although several grass species are suitable to be used for phytoremediation purposes (Table 2), considering the particularities mentioned above, the use of non-hyperaccumulator species to lower trace element concentrations in the environment to acceptable levels often takes a long time, in the order of decades or even centuries (Vangronsveld et al., 2009). For example, 22 and 26 years would be needed to reduce the pseudo-total Cd concentration of 3.6 mg kg**^–^**^1^ soil to 0.5 mg kg**^–^**^1^ soil (background reference values for São Paulo State, Brazil), using *P. maximum* and *B. decumbens*, respectively (Rabêlo et al., 2020a). Toxicity induced by trace elements compromises not only the capacity of the plant to remove trace elements, but also its capacity to stabilize polluted soils (Chen and Wong, 2006; Gao et al., 2020). Therefore, there remains a general need for the improvement of extraction and stabilization efficiencies. Some strategies can enhance phytoextraction and phytostabilization efficiencies by changing the plant availability of trace elements and/or increasing the trace elements tolerance of the plants, such as (i) the optimization of agronomic practices (e.g., by supplying nutrients and beneficial); (ii) optimizing the plant-microorganism interactions; (iii) changing the plant growth conditions (e.g., using soil amendments); (iv) by genetic breeding to overcome the disadvantages of phytoremediation (Salt et al., 1998; Koźmińska et al., 2018; DalCorso et al., 2019).

### Nutrients and Beneficial Elements Supply

In addition to improving plant growth, an appropriate supply of nutrients [nitrogen (N), P, potassium (K), Calcium (Ca), Mg, S, boron – B, chlorine (Cl), Cu, Fe, Mn, molybdenum (Mo), Ni, and Zn] and beneficial elements [cobalt (Co), sodium (Na), selenium (Se), and silicon (Si)], can also alleviate different growth stresses, including trace element-induced stress (Sarwar et al., 2010). Depending on the situation (e.g., plant species, growth conditions, and low level of pollution by trace elements), optimizing the nutritional status can contribute to improving phytoextraction or phytostabilization efficiencies. Many studies have been conducted to assess mainly the effects of N, S, and Si supplementation on the tolerance of grasses adopted for trace element phytoremediation.

Nitrogen can alleviate trace element toxicity by increasing chlorophyll contents and thus enhancing the photosynthetic capacity, supporting the higher synthesis of N-containing metabolites like amino acids and derivatives, GSH, and PCs, and by enhancing the activities of antioxidative enzymes (Sharma and Dietz, 2006; Sarwar et al., 2010; Singh et al., 2016), but for each trace element, these effects depend on specific combinations of ammonium (NH_4_^+^) and nitrate (NO_3_^–^). The supply of N as NO_3_^–^ was reported to support Cu phytostabilization, whilst the combination of NH_4_^+^ (30%) and NO_3_^–^ (70%) enhanced the activity of the antioxidative system of *P. maximum* and increased Cu phytoextraction (Souza Junior et al., 2019). The negative effects of Cd on photosynthesis and the balance between oxidants and antioxidants in *P. maximum* were also attenuated by the supply of combinations of NH_4_^+^ (50%) and NO_3_^–^ (50%), which resulted in higher Cd phytoextraction (de Sousa Leite and Monteiro, 2019a,b).

Similarly, S can also alleviate trace element toxicity by promoting the synthesis of S-containing metabolites like GSH and PCs, enhancing the activity of the AsA-GSH cycle, and regulating ethylene signaling, among other factors (Sarwar et al., 2010; Singh et al., 2016). An appropriate S supply allowed higher Cd translocation from the roots to the stems in *P. maximum* (Rabêlo et al., 2018c), as well as higher GSH and PCs synthesis, and improved the activities of enzymes of the AsA-GSH cycle, which is essential to lower Cd phytotoxicity and tiller mortality rate in grasses, which contributed to a higher Cd phytoextraction efficiency (Rabêlo et al., 2017a,b, 2018b). *Panicum maximum* appropriately provided with S was more tolerant to Cu-exposure (Gilabel et al., 2014).

Silicon can alleviate trace element toxicity through several mechanisms that include the regulation of trace element uptake and root-to-shoot translocation, modulation of the cation binding capacity of the cell walls, increase of enzymatic (e.g., SOD, APX, and DHAR) and non-enzymatic (e.g., AsA and GSH) antioxidants, and the complexation or co-precipitation of trace element ions with Si in the cytoplasm, followed by sequestration of the trace elements in the vacuoles (Pilon-Smits et al., 2009). This is of particular importance in monocots that show high Si accumulation (10–15%) (Hodson et al., 2005). Supplementation of Si lowered Cu translocation from the roots to the shoots in *P. maximum*, alleviating the Cu toxicity in the leaves, which improved the biomass production and allowed the successful harvesting of the aboveground biomass (Vieira Filho and Monteiro, 2020). Silicon also appears to play an important role in Cu tolerance in *S. densiflora*, not by avoiding its uptake by roots, but *via* lowering the Cu translocation from the roots to the leaves, resulting in a general decrease of Cu-induced deleterious effects on the photosynthetic apparatus (Mateos-Naranjo et al., 2015). Evaluating the influence of CaO-activated Si-based slag amendment on growth and Cd, Cr, Cu, Pb, and Zn uptake of *V. zizanioides*, Mu et al. (2019) found that the beneficial effect of Si depends on the dose and trace element speciation.

### Plant–Microorganism Interactions

The interaction between plant roots and microorganisms can support phytostabilization through the association with AM fungi, and phytoextraction through the association with plant growth-promoting bacteria (PGPBs) (DalCorso et al., 2019). This section focuses on the possible role of PGPB since the effects of AM fungi on trace element uptake and plant tolerance to trace elements were already described above. Plant growth-promoting bacteria, such as phosphate and potassium solubilizers, free-living N_2_-fixing bacteria, and rhizobia, can promote plant growth and increase the biomass yield by the production of phytohormones [such as indoleacetic acid (IAA)], lowering the stress originating from ethylene production [by lowering the levels of 1-aminocyclopropane-1-carboxylic acid (ACC) due to ACC-deaminase (EC 3.5.99.7) activity], or by improving the plant nutritional status through N_2_ fixation, phosphate-solubilization, or siderophore production (Sessitsch et al., 2013). Plant growth promotion plays a major role in the extraction and removal of trace elements since the enhancement of biomass production results in a higher overall trace element yield (amount of phytoextracted trace element) (McGrath and Zhao, 2003; Sessitsch et al., 2013). The influence of 14 strains of Cd/Zn-resistant rhizosphere bacteria (isolated from Poaceae and woody trees/shrubs) was evaluated on the growth and trace elements accumulation of *F. pratensis* (Becerra-Castro et al., 2012). Almost all strains promoted the growth of this grass. In the same way, the potential use of Cd-resistant bacteria in *P. purpureum* × *americanum* was evaluated, and plants inoculated with *Micrococcus* sp. showed higher shoot biomass and Cd accumulation compared with non-inoculated plants after six months of growth (Wiangkham and Prapagdee, 2018). In addition to plant growth promotion, bacteria have been reported to have beneficial effects on plant stress tolerance, likely due to the enzyme ACC deaminase, leading to lower levels of stress-induced ethylene in the plant (Sessitsch et al., 2013). The bacterium *Pseudomonas asplenii* AC was genetically transformed to express a bacterial gene encoding the enzyme ACC deaminase, and both native and transformed bacteria were tested in *P. australis*. The inoculation of seeds with the transformed *P. asplenii* AC significantly increased the seed germination and enabled the plants (shoot and roots) to reach a greater size than non-inoculated plants when exposed to Cu (Reed et al., 2005). The cloning and expression of an ACC deaminase gene in an *Azoarcus* strain provided a recombinant strain that protected inoculated rice plants against Cd stress and increased the Cd concentration in the shoots (Fernández-Llamosas et al., 2020). The use of bacteria to support plants employed in trace element phytoremediation has proved to be advantageous, and it can be much more explored since up to 99% of the soil microbial taxa are yet to be cultured and investigated (Pham and Kim, 2012).

### Utilization of Soil Amendments in Phytoremediation

The mobility and bioavailability of trace elements in the soil can be changed by the addition of either organic or inorganic amendments (Vangronsveld et al., 1995, 1996; Wiszniewska et al., 2016). Organic amendments are considered especially effective for Cr stabilization, whereas in the case of other trace elements (As, Cu, Pb, Cd, and Zn) they may have both positive and negative effects (Kumpiene et al., 2008). The addition of humic acid did not change the Cu and Zn bioavailability in soil affected by mining activities, but its use reduced Cu and Zn translocation in *V. zizanioides*, so that the combined use of this species with humic acid at 10–20 g kg^–1^ can be an effective strategy for both Cu and Zn phytostabilization in some mine soils (Vargas et al., 2016). The effects of two organic amendments (organic fertilizer – pig slurry and plant ash and sewage sludge) on the stabilization of Cd, Cu, Pb, and Zn in acidic soil established with *P. americanum* × *purpureum*, *Euchlaena mexicana*, or *S. dochna* were evaluated, and the use of 5% organic fertilizer only or combined with 5% sewage sludge decreased the trace element bioavailability and the trace element concentrations in the shoots (Zhang et al., 2016).

Some organic amendments, such as biochar and ethylenediaminetetraacetic acid (EDTA) were reported to increase phytoextraction instead of promoting trace elements phytostabilization (references). Although this result is desirable, caution is needed for its use in assisted grass growth since such plants are not efficient in performing phytoextraction in soils heavily polluted by trace elements. Therefore, the effects of soil amendments should be evaluated case-by-case (Wiszniewska et al., 2016). Combined use of biochar and PGPB was found to increase Cd uptake by 412% and the Cd BCF by 403% in *V. zizanioides* compared with the non-amended control (Wu et al., 2019). The EDTA application increased the Cr concentration in the roots and shoots, the Cr BCF, and Cr TF in *P. americanus* × *purpureum*, leading to the improper functioning of PSII, and a lower biomass yield due to Cr toxicity (Ram et al., 2019). These findings show that although organic amendments are generally considered as effective for Cr stabilization (Kumpiene et al., 2008), the opposite effect can occur due to differences in the growth medium.

The use of inorganic soil amendments such as lime, clay minerals, phosphorus-bearing materials, Mn oxides, and alkaline materials often decreases the trace element bioavailability, enhancing the trace element phytostabilization (Kumpiene et al., 2008; Wiszniewska et al., 2016). Liming was reported to be necessary to ensure the persistence of the grass *C. dactylon* grown in Cu-polluted soils, by lowering the Cu accumulation in its leaves due to a decreased plant availability of Cu produced by the increase of the soil pH (Shutcha et al., 2010). Similarly, the trace element-immobilizing agents, namely, cement, slag, phosphate rock, bitumen, Fe- and aluminum (Al)-gels, and synthetic Mn oxide (δ-MnO_2_) reduced the bioavailability of Cd and Pb in a soil covered with *E. stagninum* (Aboulroos et al., 2006). From the studies concerning organic and inorganic soil amendments, it is clear that the use of soil amendments for assisting the growth of grasses can improve both the phytoextraction and phytostabilization of trace elements, but the results largely depend on the soil conditions, the level of pollution, trace element speciation, plant species, etc. Therefore, the effects of soil amendments must be evaluated case-by-case, especially considering the fact that high trace element bioavailability induced by soil organic amendments such as biochar and EDTA can decrease plant growth and biomass yield, and lower phytoextraction efficiency. Moreover, limited information is available concerning the long-term effects of soil amendments on the soil microbiota and the metabolism of plants used for trace element phytoremediation, as well as on the entire ecosystem (Kumpiene et al., 2008; Vangronsveld et al., 2009; Wiszniewska et al., 2016; DalCorso et al., 2019).

### Plant Genetic Breeding for Phytoremediation Applications

The ability of plants to take up, translocate, and transform trace elements, as well as to limit their toxicity, may be significantly enhanced *via* genetic engineering (for a comprehensive review see Koźmińska et al., 2018). Although genetic transformation has been developed or improved for several grass species during the last decades (Wang and Ge, 2006), this technology is still poorly explored in the context of trace element phytoremediation. Only a few studies employed genetic manipulation to improve the desirable characteristics of grasses for trace element phytoremediation (Czakó et al., 2005, 2006; Taghizadeh et al., 2015).

Czakó et al. (2005) assessed the transfer of gene encoding enzymes for the breaking down of organomercurials [Organomercurial lyase (MerB, EC 4.99.1.2)] and the reduction of Hg^2+^ to Hg^0^ [Mercuric reductase (MerA) (EC 1.16.1.1)] into the *S. alterniflora*. They reported that all but one transgenic line contained both *MerB* and *MerA* sequences, demonstrating that the co-introduction into *S. alterniflora* of two genes from separate *Agrobacterium* strains is possible. A wild-type callus of *S. alterniflora* was sensitive to phenylmercuric acetate (PMA) at 50 μmol L^–1^ and to HgCl_2_ at 225 μmol L^–1^, but the transgenic line #3 was resistant to PMA, whereas the transgenic line #7 showed resistance to HgCl_2_ (up to 500 μmol L^–1^) (Czakó et al., 2006). Plant breeding by *in vitro* culture to improve the tolerance and accumulation of Pb in *C. dactylon* was evaluated and the results of the study revealed the occurrence of somaclonal variation *via* somatic embryogenesis and organogenesis of *C. dactylon* cultures with a frequency of 33% (Taghizadeh et al., 2015). Some *in vitro* derived plants had higher Pb concentrations in the shoot, but there were also some regenerates with lower Pb concentrations in the shoot and some without any changes in Pb concentrations compared with the control.

Although some progress has been achieved after a long-term *in vitro* selection of trace element-tolerant clones during the last years, modifications involving genome editing and genome engineering to insert gene(s) responsible for trace element transport and homeostasis or defense responses to oxidative stress can be a more efficient strategy (Koźmińska et al., 2018). Numerous pathways can be manipulated to increase the trace elements uptake, translocation, and accumulation in plant tissues. However, to increase trace elements uptake by grasses it is necessary to increase their tolerance, otherwise, the phytotoxicity induced by trace elements certainly will lead to the death of the plant. The genes that are currently widely used to manipulate trace element metabolism in plants are those that encode for transporters of trace element ions and trace element-binding ligands (e.g., *GSH1* and *PCS*) (Yadav, 2010; Koźmińska et al., 2018). However, to the best of our knowledge, there is no study about this in grasses used for phytoremediation.

## Advantages and Disadvantages of the Use of Grasses for Trace Element Phytoremediation

The use of grasses for trace element phytoremediation has advantages and disadvantages compared with the use of other plant species; some particularities are described below.

### Advantages

**FIGURE 6:**
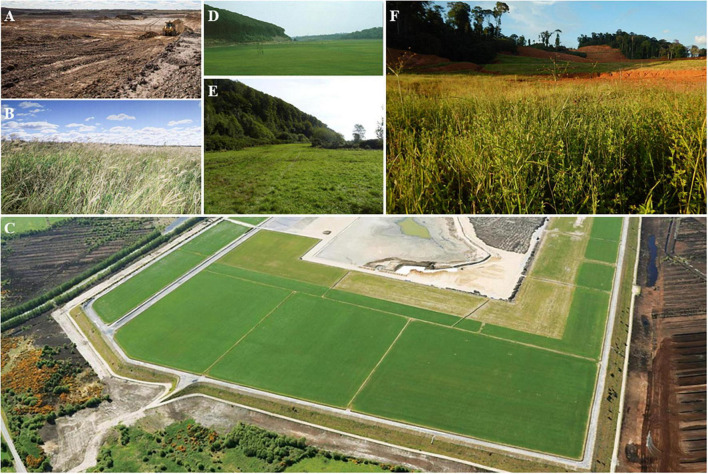
Rehabilitation of mining areas in Australia **(A,B)**, Ireland **(C–E)**, and Guyana **(F)** by using grasses (grass species not specified). **A:** Commodore Coal Mine before rehabilitation (Minerals Council of Australia [MCA], 2016); **B:** Commodore Coal Mine after rehabilitation (Minerals Council of Australia [MCA], 2016); **C:** Tailings management facility rehabilitation at Lisheen Mine (Courtney, 2018); **D:** Shelton Abbey rehabilitated tailings site after shortly seeding in 1982 (Courtney, 2018); **E:** Shelton Abbey rehabilitated tailings site after seeding in September 2016 (Courtney, 2018); **F:** Rehabilitation of mining sites located in the heart of the Amazon rainforest (https://esperancegoldmine.com/index.php/en/rehabilitation-of-mine-sites/).

(i) Grasses colonize land from cold regions to humid wetlands in the tropics spontaneously (Zeven and de Wet, 1982) and can be efficiently used for phytoextraction of mildly polluted agricultural soils (Figure 5), phytostabilization, or phytofiltration of trace elements (Table 2) during the rehabilitation of polluted areas (Figure 6);
(ii) Perennial grasses exposed to trace elements can sprout again after shoot harvest (Gilabel et al., 2014; Rabêlo et al., 2017a), avoiding the need of replanting after each harvest;
(iii) Grasses often deliver high biomass yield (sometimes more than 20 t DW ha^–1^ yr^–1^; Rabêlo et al., 2018a) that can be used after harvesting as a source for bioenergy (biogas, bioethanol, bio-oil) (Balsamo et al., 2015), thus with added economical value and eventually, also for the recovery of trace elements;
(iv) Growing and harvesting grasses do not require expensive equipment or highly qualified staff.

### Disadvantages

(i) The growth and biomass yield of grasses (mainly of those grasses with high biomass yield) can slow down or even stop due to water deficit (Ings et al., 2013) and low temperatures (Lewandowski et al., 2003), which compromises the phytoremediation efficiency;
(ii) The use of grasses for phytoremediation is limited to the upper soil layer. The rooting depth varies with the grass species used, but often a major fraction of the root system is concentrated in the upper 0–15 cm (Gist and Smith, 1948);
(iii) Many grass species can only be used in sites polluted with one or two trace elements, which restricts their use in multi-element polluted sites (Table 2).

## General Conclusion and Perspectives

Undoubtedly, grasses have a tremendous potential to stabilize trace elements in soils, sediments, and wastewater. However, much more studies employing molecular approaches are required to better understand the processes involved in the uptake, transport, and accumulation of trace elements. Several types of grass have been investigated, but only a limited number of transporters of As, Cd, Cu, and Zn have been identified in just a few species. The expression of genes encoding for trace element transporters is species- and tissue-specific. Understanding the role of each trace element transporter in grasses is essential to optimize the phytoremediation of trace elements. Studies aiming to identify trace element transporters and to elucidate mechanisms related to uptake, transport, and accumulation of trace elements should be conducted, especially for Cr, Hg, Ni, and Pb. The most existing studies aiming to better understand the physiological processes of the induction of trace element toxicity and how grasses cope with this toxicity are based on acute trace elements exposure. This makes it difficult to link the results to tolerance mechanisms since, in the first stage of trace element exposure, a sequence of signaling events occurs to adapt the plant to the stress. Under field conditions, however, exposure to a trace element is chronic which often provokes a range of responses very different from those observed in case of acute exposure (Gallego et al., 2012). Therefore, studies need to be conducted under more realistic scenarios, since most grasses do not survive for a long time when exposed to high doses of different trace elements. A range of grass species may be suitable for the phytoextraction of mildly polluted agricultural soils or phytostabilization of soils with low-to-moderate concentrations of trace elements, and only a few species are appropriate for phytofiltration of trace elements. The efficacies of grasses for phytoextraction or phytostabilization can be improved using amendments and microorganisms supporting plant growth, but the adoption of such strategies should be assessed case-by-case. Increases in the trace element uptake by plants instead of better trace element phytostabilization can occur and *vice versa* due to the specificity of trace elements, amendment/microorganisms, and plant interactions. Genetic manipulation, which is another alternative to increase the efficiencies of phytoextraction and phytostabilization of trace elements, has been largely neglected due to its genetic complexity and the associated difficulties encountered during conventional breeding of grasses (Wang and Ge, 2006), leaving an important gap open that might be explored during the next years. The rehabilitation of trace element-polluted sites by using only grasses was proven to be an efficient strategy (Minerals Council of Australia [MCA], 2016; Courtney, 2018), but the cultivation of grasses associated with other plants such as legumes and trees can accelerate site rehabilitation and is more adequate from an ecological point of view (Kumar et al., 2017; Wen et al., 2018).

## Author Contributions

FHSR conceived the project and wrote the manuscript. JV, AJMB, AVDE, and LRFA reviewed the manuscript by rewriting, discussing, and commenting. All the authors contributed to the article and approved the submitted version.

## Conflict of Interest

The authors declare that the research was conducted in the absence of any commercial or financial relationships that could be construed as a potential conflict of interest.

## Publisher’s Note

All claims expressed in this article are solely those of the authors and do not necessarily represent those of their affiliated organizations, or those of the publisher, the editors and the reviewers. Any product that may be evaluated in this article, or claim that may be made by its manufacturer, is not guaranteed or endorsed by the publisher.
